# Pterosaur dietary hypotheses: a review of ideas and approaches

**DOI:** 10.1111/brv.12431

**Published:** 2018-06-07

**Authors:** Jordan Bestwick, David M. Unwin, Richard J. Butler, Donald M. Henderson, Mark A. Purnell

**Affiliations:** ^1^ School of Geography, Geology and the Environment University of Leicester Leicester LE1 7RH U.K.; ^2^ School of Museum Studies University of Leicester Leicester LE1 7RF U.K.; ^3^ School of Geography, Earth and Environmental Sciences University of Birmingham Birmingham B15 2TT U.K.; ^4^ Royal Tyrrell Museum of Palaeontology, P.O. Box 7500 Drumheller Alberta, T0J 0Y0 Canada

**Keywords:** pterosaur, ecosystem, diet, interpretations, qualitative, quantitative, evidence, consensus, comparative anatomy

## Abstract

Pterosaurs are an extinct group of Mesozoic flying reptiles, whose fossil record extends from approximately 210 to 66 million years ago. They were integral components of continental and marginal marine ecosystems, yet their diets remain poorly constrained. Numerous dietary hypotheses have been proposed for different pterosaur groups, including insectivory, piscivory, carnivory, durophagy, herbivory/frugivory, filter‐feeding and generalism. These hypotheses, and subsequent interpretations of pterosaur diet, are supported by qualitative (content fossils, associations, ichnology, comparative anatomy) and/or quantitative (functional morphology, stable isotope analysis) evidence. Pterosaur dietary interpretations are scattered throughout the literature with little attention paid to the supporting evidence. Reaching a robustly supported consensus on pterosaur diets is important for understanding their dietary evolution, and their roles in Mesozoic ecosystems. A comprehensive examination of the pterosaur literature identified 314 dietary interpretations (dietary statement plus supporting evidence) from 126 published studies. Multiple alternative diets have been hypothesised for most principal taxonomic pterosaur groups. Some groups exhibit a high degree of consensus, supported by multiple lines of evidence, while others exhibit less consensus. Qualitative evidence supports 87.3% of dietary interpretations, with comparative anatomy most common (62.1% of total). More speciose groups of pterosaur tend to have a greater range of hypothesised diets. Consideration of dietary interpretations within alternative phylogenetic contexts reveals high levels of consensus between equivalent monofenestratan groups, and lower levels of consensus between equivalent non‐monofenestratan groups. Evaluating the possible non‐biological controls on apparent patterns of dietary diversity reveals that numbers of dietary interpretations through time exhibit no correlation with patterns of publication (number of peer‐reviewed publications through time). 73.8% of dietary interpretations were published in the 21st century. Overall, consensus interpretations of pterosaur diets are better accounted for by non‐biological signals, such as the impact of the respective quality of the fossil record of different pterosaur groups on research levels. That many interpretations are based on qualitative, often untestable lines of evidence adds significant noise to the data. More experiment‐led pterosaur dietary research, with greater consideration of pterosaurs as organisms with independent evolutionary histories, will lead to more robust conclusions drawn from repeatable results. This will allow greater understanding of pterosaur dietary diversity, disparity and evolution and facilitate reconstructions of Mesozoic ecosystems.

## I. INTRODUCTION

**Figure 1:**
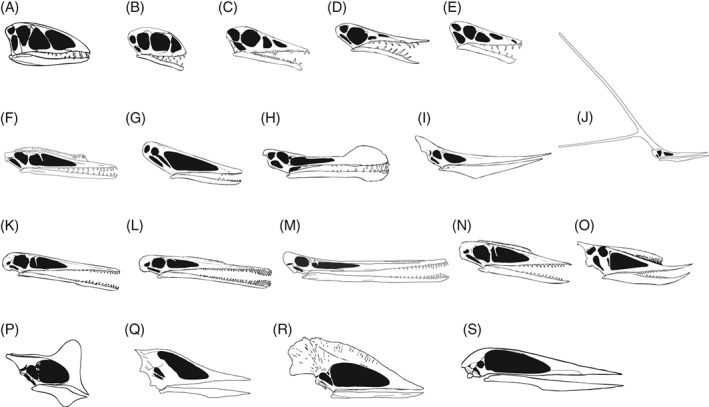
Examples of pterosaur skull and dental diversity from each principal group in this study. Skulls traced or redrawn from Unwin (2006) and references therein, unless otherwise stated. (A) The basal‐most pterosaur *Dimorphodon macronyx*; (B) the anurognathid *Anurognathus ammoni*; (C) the campylognathoidid *Eudimorphodon ranzii*; (D) the rhamphorhynchine *Rhamphorhynchus muensteri*; (E) the scaphognathinine *Scaphognathus crassirostris*; (F) the basal monofenestratan *Darwinopterus modularis,* traced from Lü *et al.* (2010); (G) the istiodactylid *Istiodactylus latidens*; (H) the ornithocheirid *Ornithocheirus mesembrinus*; (I) the pteranodontid *Pteranodon longiceps*; (J) the nyctosaurid *Nyctosaurus gracilis*, redrawn from Bennett (2003a); (K) the basal ctenochasmatoid *Pterodactylus antiquus*; (L) the ctenochasmatid *Gnathosaurus subulatus*; (M) the lonchodectid *Feilongus* sp., redrawn from Wang *et al.* (2005); (N) the basal dsungaripteroid *Germanodactylus cristatus*; (O) the dsungaripterid *Dsungaripterus weii*; (P) the tapejarid *Tapejara wellnhoferi*; (Q) the chaoyangopterid *Shenzhoupterus chaoyangensis,* traced from Lü *et al.* (2008a); (R) the thalassodromid *Tupuxuara leonardii*; (S) the azhdarchid *Zhejiangopterus linhaiensis*. Skulls not drawn to scale.

Pterosaurs are an extinct clade of flying Mesozoic reptiles, with a 150‐million‐year fossil record from the Late Triassic to the latest Cretaceous (Wellnhofer, 1991; Chatterjee & Templin, 2004; Unwin, 2006; Butler, Benson & Barrett, 2013). The current number of described pterosaur species is around 190, and the clade exhibits wide morphological disparity (Fig. 1) (Prentice, Ruta & Benton, 2011; Butler *et al*., 2012; Foth, Brusatte & Butler, 2012; Hyder, Witton & Martill, 2014), with wingspans ranging from 40 cm to 10 m (Hone & Benton, 2008; Benson *et al*., 2014). Pterosaur research has advanced rapidly during the last three decades (Barrett *et al*., 2008; Hone, 2012; Witton, 2013), with new finds of fossil Lagerstätten in Brazil and China yielding numerous well‐preserved pterosaur specimens. These have formed the basis of large numbers of new taxa and greatly increased understanding of pterosaur evolution (Barrett *et al*., 2008; Lü & Bo, 2011; Hone, 2012; Dean, Mannion & Butler, 2016). Aerodynamic models of bone strength analyses and wing‐loading forces have revealed that pterosaurs potentially flew with high manoeuvrability (Palmer, 2011; Habib & Hall, 2012; Habib & Witton, 2013), and likely utilised energy‐efficient quadrupedal take‐offs (Habib, 2008). Pterosaur phylogeny (Kellner, 2003; Unwin, 2003; Lü *et al*., 2010; Andres, Clark & Xu 2014), ground‐based behaviours elucidated from trace fossils (ichnofossils) (Hwang *et al*., 2002; Xing *et al*., 2012; Fiorillo *et al*., 2015) and reproductive biology (Chiappe *et al*., 2004; Grellet‐Tinner *et al*., 2007; Lü *et al*., 2011a; Wang *et al*., 2015, 2017) have also seen major advances.

One area of pterosaur research lagging behind, however, is understanding of their dietary ecology (Unwin & Henderson, 2002; Hone, 2012; Hone *et al*., 2015a). Diet, as used here, refers to the food items typically consumed by a species. A range of pterosaur diets have been proposed, including carnivory, piscivory, insectivory, durophagy and filter‐feeding (Seeley, 1901; Wellnhofer, 1991; Unwin, 2006; Veldmeijer, Witton & Nieuwland, 2012; Witton, 2013). Pterosaurs were significant components of many Mesozoic ecosystems, and helped shape the evolution of Mesozoic food webs. Reaching a consensus on diets for major pterosaur groups is therefore essential for understanding pterosaur dietary evolution and for reconstructing Mesozoic ecosystems (Unwin & Henderson, 2002). Pterosaur dietary hypotheses and interpretations are scattered across the literature, and whilst popular texts have summarised interpretations (Wellnhofer, 1991; Unwin, 2006; Veldmeijer *et al*., 2012; Witton, 2013, 2018), there is no single synthesis of the evidence supporting these interpretations.

The evidence underpinning dietary interpretations can be broadly categorised as qualitative or quantitative, although categories are not always mutually exclusive. Qualitative lines of evidence often rely solely on inferences drawn from direct or indirect morphological comparisons with modern organisms (Unwin & Henderson, 2002; Veldmeijer, Signore & Bucci, 2007), supported by an assumption that morphologically similar structures indicate some level of functional convergence (Witton & Naish, 2008). Dentitions and skull morphologies are most commonly compared with extant analogues, because these are the parts of the body most directly involved in feeding (Fastnacht, 2005; Ősi, 2011).

Qualitative evidence also includes associations of potential food items with pterosaur fossils. Of these, specimens with remains of other organism(s) in their gut and throat (content fossils) are interpreted as direct evidence of diet (Wild, 1984; Hone *et al*., 2015a; Witton, 2018). Evidence from coprolites (fossilised faeces) falls into a similar category, but determining producers of coprolites can be challenging. Other studies infer diet from general associations with taxa from the same stratigraphic interval and/or depositional environments as the preserved pterosaur specimens (Kellner, 2003; Chatterjee & Templin, 2004; Tütken & Hone, 2010). Finally, ichnofossils are used to infer foraging behaviours and habitat preferences, leading to dietary interpretations (Lockley & Wright, 2003; Mazin *et al*., 2003; Fiorillo *et al*., 2015).

During the last 30 years, dietary analysis of extinct animals has become increasingly quantitative, allowing explicit hypothesis testing and robust, repeatable conclusions (Lauder, 1995; Ferry‐Graham, Bolnick & Wainwright, 2002; Veldmeijer *et al*., 2007; Anderson *et al*., 2011). Quantitative functional morphology methods do not provide direct evidence of actual food items, but of feeding and/or foraging behaviours that can be used to infer more or less plausible dietary hypotheses (Amiot *et al*., 2010; Bright, 2014). Finite element analysis (FEA) applies external forces to digital anatomical reconstructions and calculates resultant stress and strain distributions (Rayfield *et al*., 2001; Fastnacht, 2005; Anderson *et al*., 2011). Skull reconstructions of the pterosaur *Pteranodon*, for example, show that it experienced relatively low stresses and strains during jaw closure, indicating that it could effectively seize prey by rapid jaw closure (Fastnacht, 2005).

Stable isotope analyses of carbon (^13^C/^12^C) and oxygen (^18^O/^16^O) ratios from bone apatite and tooth enamel can reveal whether animals inhabited terrestrial or marine environments, allowing some limited dietary inferences to be drawn (Tütken & Hone, 2010).

We present the first comprehensive synthesis of pterosaur dietary interpretations. For each interpretation, we identify dietary statements and underpinning evidence categories and evaluate how robustly supported different dietary interpretations are within, and between, key pterosaur groups. This provides the basis for a discussion of biological and non‐biological influences on apparent pterosaur dietary diversity.

## II. METHODS

### (1) Phylogenetic frameworks

**Figure 2:**
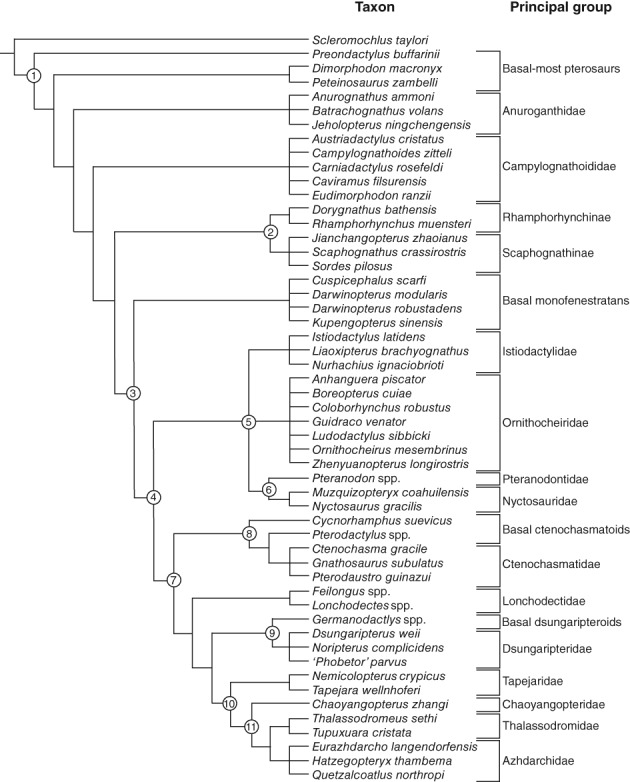
Pterosaur phylogeny used as a framework for this study, based on Unwin (2003) and Lü *et al.* (2010). Principal groups denote the order of discussion in Section IV from the base of the Pterosauria. Taxa within the phylogeny are explicitly mentioned herein and do not necessarily denote all members of respective groups. Branch lengths do not infer phylogenetic distances. Polytomies are given for unknown phylogenetic relationships. The avemetatarsalian *Scleromochlus taylori* is included as an outgroup. Nodes: 1, Pterosauria; 2, Rhamphorhynchidae; 3, Monofenestrata; 4, Pterodactyloidea; 5, Ornithocheiroidea; 6, Pteranodontoidea; 7, Lophocratia; 8, Ctenochasmatoidea; 9, Dsungariptoidea; 10, Azhdarchoidea; 11, Neoazhdarchia.

Pterosauria is defined as the most recent common ancestor of *Preondactylus buffarinii* and *Quetzalcoatlus northropi* and all its descendants (Fig. 2) (Kellner, 2003; Unwin, 2003). Pterosauromorpha comprises Pterosauria and ornithodiran archosaurs which share more recent common ancestors with Pterosauria than with its sister group, Dinosauromorpha (Nesbitt, 2011). *Scleromochlus taylori* from the early Late Triassic (around 230 Ma) of Scotland has been considered as a basal pterosauromorph (Padian, 1984), but the position of *Scleromochlus* within Ornithodira is unclear (Benton, 1999; Nesbitt, 2011).

Phylogenetic analyses of Pterosauria (Kellner, 2003; Unwin, 2003; Lü *et al*., 2010; Andres *et al*., 2014), show agreement in identifying approximately 20 principal groups, each sharing consistent features of the jaws, dentition (where present), and cranial and postcranial anatomy (Figs 1 and 2). Some of these groups are clades, whilst others are grades of morphologically similar taxa. These groups form the taxonomic units used herein.

The taxonomic contents of these groups exhibit similarities between published phylogenies, but they are not universally agreed upon. Three distinct phylogenies are therefore used to examine the impact of phylogenetic uncertainty when considering dietary hypotheses. These phylogenies are referred to as ‘Unwin’, ‘Kellner’ and ‘Andres’ after the authors of the initial iterations of these data sets. Herein, the Unwin phylogeny represents a composite tree of Unwin (2003) and Lü *et al*. (2010) (Fig. 2). The Kellner phylogeny is a composite tree of Kellner (2003), Wang *et al*. (2009), Wang *et al*. (2012) and Rodrigues *et al*. (2015) (see online Appendix S1, Fig. S1). The Andres phylogeny is from Andres *et al*. (2014; see online Fig. S2). Where sets of phylogenies used to construct a tree conflict, relevant taxa are collapsed into a polytomy. Whilst the preferential selection of any one of these phylogenies is to some extent arbitrary, the Unwin phylogeny is primarily used herein because it exhibits the highest stratigraphic congruence (Andres, 2015). Labelled principal groups in the Unwin phylogeny (Fig. 2) thus denote the order of Section IV. Further information on phylogenies is included as online Supporting Information (see online Appendix S1).

### (2) Dietary and evidential categories

We used seven principal dietary categories: (*i*) insectivory: insects and unarmoured terrestrial invertebrates; (*ii*) piscivory: fish and other nektonic organisms such as cephalopods (Hone *et al*., 2015a); (*iii*) carnivory: terrestrial vertebrates (predation and/or scavenging); (*iv*) durophagy: consumption of organisms with hard shells or armour (Crofts & Summers, 2014), including seeds and non‐planktonic aquatic crustaceans and molluscs; (*v*) herbivory/frugivory: fruits and plant matter; (*vi*) filter‐feeding on planktonic crustacean, mollusc and/or fish larvae; (*vii*) generalists: where authors explicitly mention this dietary category.

We assign the evidence used to support dietary interpretations to six categories: (*i*) content fossils, including coprolites; (*ii*) spatiotemporal associations with taxa and/or depositional environments; (*iii*) ichnofossils; (*iv*) comparative anatomy; (*v*) functional morphology; (*vi*) stable isotope analysis.

### (3) Data sets

#### (a) *Pterosaur diets*

The primary data set for this study is a compilation of interpretations, each comprising a statement regarding the diet of a taxon that either is one of the recognised principal taxonomic groups, or falls within one of those groups. Each interpretation was unambiguously assigned to one dietary category and to one evidence category. Dietary statements without supporting evidence were excluded.

Dietary interpretations were compiled from the literature. Publications citing previous interpretations but lacking novel data or reasoning were excluded. One hundred and twenty six publications contained at least one novel dietary interpretation. When a publication identified the same dietary and evidence categories for multiple taxa within the same principal group, this was treated as a single novel interpretation. When a publication provided a single dietary statement for a single group but supported it with more than one evidential category, this was tabulated under each evidence category.

Differences in the taxonomic content of principal groups between the phylogenies led to differences in the numbers of identified interpretations. For example, a publication assessing diet for two species from the same principal group in one phylogeny was treated as one interpretation. If these two species then fell within different groups in a different phylogeny, this interpretation was counted twice, once for each group. As a result, 314 interpretations were identified for the Unwin phylogeny, 311 for the Kellner phylogeny and 301 for the Andres phylogeny.

Details of interpretations for each phylogeny are included as online Supporting Information (see online Appendix S1).

#### (b) *Species listing*

Valid pterosaur species were derived from Dean *et al*. (2016) and a literature review, ending in February 2017. Eight species could not be referred to any of the principal groups and were thus excluded (see online Appendix S1). The final data set comprises 180 species (see online Appendix S1).

#### (c) *Pterosaur publications from 1784 to 2017*

A data set of all pterosaur‐focused publications was compiled to estimate research effort through time, from 1784 to February 2017. The sum of all publications (1828) is likely a slight underestimate.

### (4) Analyses

Numbers of interpretations for each dietary and evidence category were summed for each principal group. Total percentages of each evidence category were calculated. To investigate possible biological and non‐biological drivers of the diversity of dietary interpretations, numbers of species from each principal group were compared to numbers of assigned dietary categories. More speciose groups are hypothesised to have greater dietary diversity because they exhibit more morphological variation (Zhou *et al*., 2017). Ornithocheiridae was by far the most speciose group with 36 species and was removed as an outlier (the second was Ctenochasmatidae with 18 species). The number of dietary groups was non‐normally distributed (Shapiro–Wilk, *P* < 0.05), and therefore a Spearman's rank correlation was used when comparing dietary groups and species counts.

Pterosaur publications were sorted into decadal bins from 1780–1789 to 2010–2017 and compared with dietary interpretations, sorted into the same time‐bins. Publications and dietary interpretations were transformed using generalised differencing to reduce the likelihood of type I errors when performing correlations on time‐series data. Correlations were then tested using Spearman's rank tests. Cumulative data on publications and dietary interpretations were plotted over the same timespan.

To assess changes in dietary consensus through time, dietary interpretations for each principal group were assigned to one of four different stages of pterosaur research history (Witton, 2013): (*i*) late 18th and 19th centuries (*N* = 3 from two publications); (*ii*) 1900–1969 (*N* = 17 from eight publications); (*iii*) 1970–1999 (*N* = 61 from 22 publications); (*iv*) 2000–present (*N* = 233 from 94 publications).

## III. RESULTS

### (1) Analysis of pterosaur dietary interpretations

**Figure 3:**
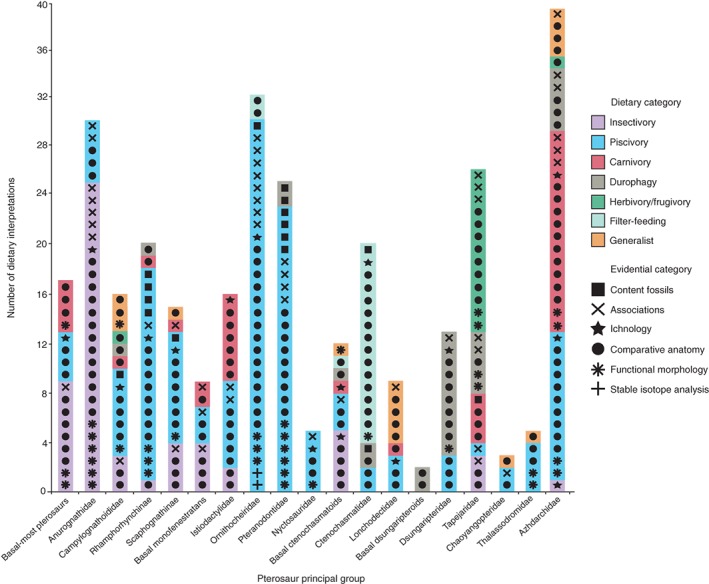
Number of pterosaur dietary interpretations for each pterosaur principal group in the Unwin phylogeny. Entries include respective dietary statements (denoted by colour) and evidential category (denoted by symbol). Each symbol with underlying colour denotes one interpretation (*N* = 314 from 126 studies). See Section II.2 for dietary and evidential category definitions. Full breakdowns of interpretations are included as online Supporting Information (see online Appendix S1).

Total numbers of dietary interpretations for each principal group (Fig. 3) show large differences, from 39 in Azhdarchidae to just two in basal dsungaripteroids (although the latter comprises just one genus). Marked disparity also occurs in numbers of hypothesised diets. For Nyctosauridae and basal dsungaripteroids, for example, only a single diet has been proposed, whereas for Campylognathoididae, basal ctenochasmatoids and Azhdarchidae there are six different diets (Fig. 3). For most groups, a single diet is supported by multiple evidential categories, with one or more other diets suggested by one or a few interpretations. For some groups, different diets are hypothesised for the same taxa within these groups, and in others different diets are associated with particular taxa (see online Appendix S1). Some groups, such as Istiodactylidae and Azhdarchidae, exhibit roughly even splits between the two most common diets, while others, such as basal ctenochasmatoids, exhibit small disparities between most‐supported and lesser‐supported diets (Fig. 3).

### (2) Analysis of categories of evidence

**Figure 4:**
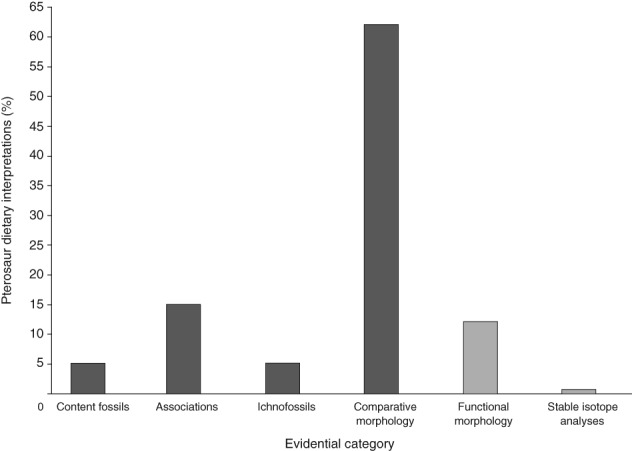
Percentage of qualitative (dark grey; content fossils, spatiotemporal associations, ichnofossils and comparative anatomy) and quantitative (light grey; functional morphology and isotope analysis) evidential categories underpinning pterosaur dietary interpretations in the Unwin phylogeny. (*N* = 314 from 126 studies).

There is a large disparity in the numbers of interpretations supported by qualitative (content fossils, associations, ichnofossils and comparative anatomy) and quantitative (functional morphology and isotope analyses) approaches, with the former supporting 87.3% of all interpretations (Fig. 4). Comparative anatomy is the most common evidence category (62.1% of total), with general associations second (15%) (Fig. 4). Interpretations based on analysis of functional morphology make up 12.1% of interpretations; content fossils and ichnofossils each make up 5.1%; stable isotope analyses make up 0.6% (Fig. 4).

### (3) Species *versus* dietary categories

**Figure 5:**
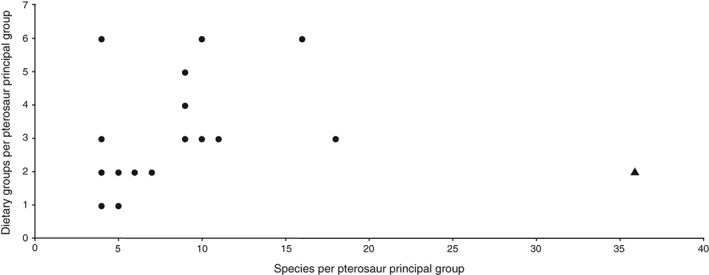
Numbers of hypothesised dietary groups per pterosaur principal group in the Unwin phylogeny is correlated with numbers of identified species per group [ρ = 0.509, *N* = 16, *P* = 0.031; Ornithocheiridae (black filled triangle) was excluded from analysis as an outlier]. Least‐squares regression (not plotted) indicates that a linear model provides a poor fit to the data (*r* ^2^ = 0.19, excluding Ornithocheiridae). Overall, more dietary hypotheses have been proposed for more speciose groups. Species assortments are included as online Supporting Information (see online Appendix S1).

There is a moderate positive correlation between pterosaur species per principal group and dietary categories for respective principal groups when Ornithocheiridae is excluded as an outlier (ρ = 0.509, *N* = 16, *P* = 0.031) (Fig. 5). When Ornithocheiridae is included, there is no significant correlation (ρ = 0.389, *N* = 17, *P* = 0.1).

### (4) Dietary consensus and phylogenies

The phylogenetic distributions of dietary interpretations for particular groups are broadly consistent among the three phylogenies (Fig. 3, see online Figs S3 and S4). For several groups one dietary interpretation is strongly supported (e.g. Anurognathidae, Pteranodontidae), with little support for others. Several groups exhibit some dietary disparity, although one interpretation remains dominant (e.g. Dsungaripteridae, Lonchodectidae). A few groups exhibit numerous interpretations, all of which receive some support (e.g. Azhdarchidae).

High congruence in terms of group definitions and content between the Unwin and Kellner trees result in similar distributions of dietary interpretations, especially for monofenestratans (Fig. 3, see online Fig. S3). There is also high congruence for Anurognathidae and Campylognathoididae, but lower congruence for ‘basal‐most pterosaurs’ (=Group A in the Kellner phylogeny) and Rhamphorhynchinae (=Rhamphorhynchidae in the Kellner phylogeny).

The distribution of interpretations for monofenestratan groups in the Andres phylogeny show high congruence with the Unwin and Kellner phylogenies (see online Fig. S4). However, ‘basal‐most pterosaurs’ and Rhamphorhynchidae in the Andres phylogeny contain substantially more interpretations than their equivalents in the other phylogenies.

### (5) Historical analyses

**Figure 6:**
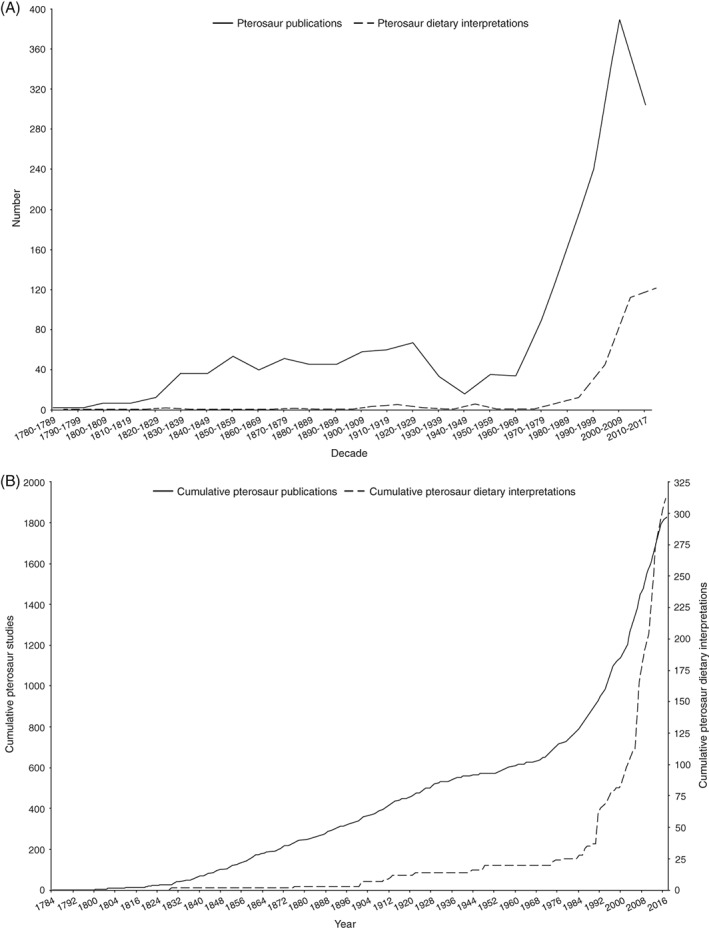
(A) Numbers of identified pterosaur publications (*N* = 1828; solid black line), and pterosaur dietary interpretations (*N* = 314; dashed black line) assorted into decadal time‐bins from 1780–1789 to the present decade, 2010–2017, ending in February 2017. (B) Cumulative pterosaur publications (left *y*‐axis, solid line) and cumulative dietary interpretations (right *y*‐axis, dashed line) each year from 1784 to 2017.

There is no correlation between numbers of pterosaur publications and dietary interpretations through time (ρ = 0.224, d.f. = 22, *P* = 0.302) (Fig. 6A). The number of publications on pterosaurs was relatively low and stable from 1830–1839 to 1920–1929 and then decreased and remained low until 1960–1969 (Fig. 6A). There was then a large increase in publications from 1970–1979 onwards (Fig. 6A). Published dietary interpretations were relatively uncommon for much of pterosaur research history, until a dramatic increase in interest from 1990–1999 onwards (Fig. 6A). This decade (2010–2017) has seen a decrease in pterosaur publications, although this may be an artefact as the decade is not yet over, and only a slight increase in dietary interpretations (Fig. 6A). The numbers of new pterosaur publications each year always exceed new numbers of dietary interpretations, except in 1991 (Fig. 6B). The earliest identified reports of hypothesised diets and evidential categories are listed in Table 1.

**Table 1:** Earliest identified reports of hypothesised pterosaur diets and evidential categories with the respective taxon/taxa.

Category	Year	Taxon	Reference
**Dietary category**			
Insectivory	1829	*Dimorphodon macronyx*	Buckland (1829)
Piscivory	1876	*Pteranodon*	Marsh (1876)
Carnivory	1913	*Istiodactylus latidens*	Hooley (1913)
Durophagy	1943	*Pteranodon*	Brown (1943)
Herbivory/frugivory	1991	*Tapejara wellnhoferi*	Wellnhofer & Kellner (1991)
Filter‐feeding	1986	*Pterodaustro*	Bakker (1986)
Generalist	2001	*Lonchodectes*	Unwin (2001)
**Evidential category**			
Content fossils	1943	*Pteranodon*	Brown (1943)
Associations	1829	*Dimorphodon macronyx*	Buckland (1829)
Ichnofossils	2002	*Haenamichnus unhangriensis* a	Hwang *et al*. (2002)
Comparative anatomy	1829	*Dimorphodon macronyx*	Buckland (1829)
Functional morphology	1974	*Pteranodon*	Bramwell & Whitfield (1974)
Stable isotope analysis	2010	*Dsungaripterus*, *Pteranodon*, Ornithocheiridae indet. Tapejaridae indet.	Tütken & Hone (2010)

a *Haenamichnus unhangriensis* is a pterosaur ichnospecies thought to have been generated by an azhdarchid.

### (6) Historical patterns in dietary interpretations

**Figure 7:**
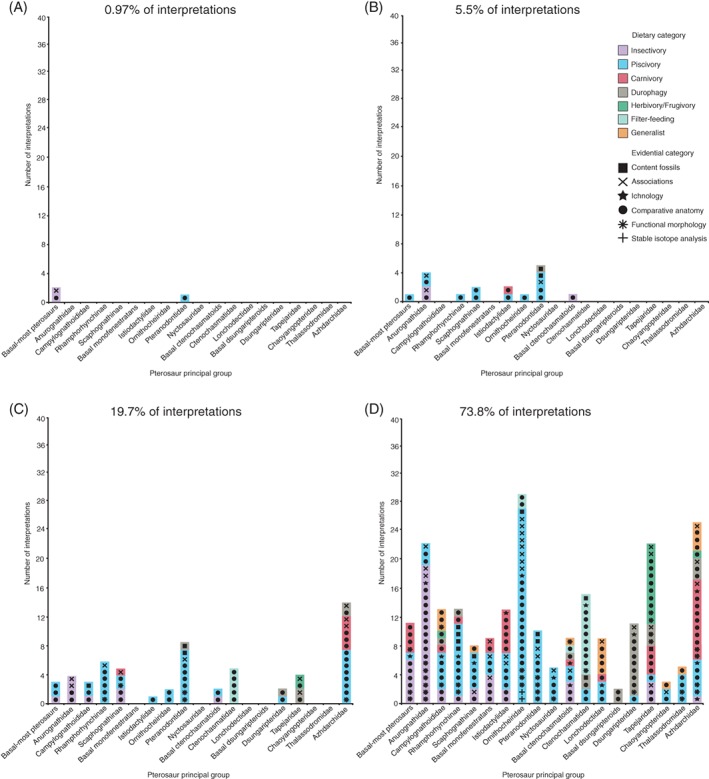
Historical precedence of pterosaur dietary interpretations with dietary statements (denoted by colour) and underpinning evidence (denoted by symbol) assorted into discrete time‐bins. (A) 19th century (*N* = 3 from two studies), (B) 1900–1969 (*N* = 17 from eight studies), (C) 1970–1999 (*N* = 61 from 22 studies), (D) 2000–present (February 2017; *N* = 233 from 94 studies). A full breakdown of these interpretations is included as online Supporting Information (see online Appendix S1).

During the 19th century, 0.97% of dietary interpretations were proposed, 5.5% were proposed between 1900 and 1969, 19.7% between 1970 and 1999, and 73.8% since 2000 (Fig. 7A–D). One or two diets were initially hypothesised for most pterosaur groups, with subsequent interpretations largely following those initial interpretations. Rhamphorhychinae and Ctenochasmatidae for example were originally hypothesised as piscivores (Fig. 7B) and filter‐feeders (Fig. 7C), respectively, which remain their most common interpreted diets to the present day (Fig. 7D). Istiodactylidae and Azhdarchidae by contrast are evenly affiliated with piscivory and carnivory through time (Fig. 7B–D).

## IV. PTEROSAUR DIETS

### (1) Non‐pterosaur pterosauromorphs

Determining the plesiomorphic dietary condition for pterosaurs is problematic, because different phylogenies recover different pterosaurs at the base of Pterosauria. Lü *et al*. (2010) for example, considers *Preondactylus buffarinii* the basal‐most pterosaur, while Kellner (2003) and Bennett (2007) consider Anurognathidae the basal group. This makes it difficult to infer which pterosaurs exhibit the plesiomorphic dietary condition. Understanding the diets of non‐pterosaur pterosauromorphs may provide a solution. At present, *Scleromochlus*, which pre‐dates the earliest known pterosaurs by around 15–20 million years (Dalla Vecchia, 2003; Nesbitt, 2011), is the only putative non‐pterosaur pterosauromorph, but its phylogenetic position is controversial. *Euparkeria capensis,* from the early Middle Triassic of South Africa, represents a close outgroup of archosaurs and may approach the plesiomorphic archosaur body plan (Senter, 2003; Nesbitt, 2011; Sookias & Butler, 2013; Sookias, 2016), and has been used as an outgroup in several phylogenetic analyses of pterosaurs (Bennett, 1996; Andres *et al*., 2014). It could be argued that understanding diets of non‐archosaur archosauriforms such as *Euparkeria* may help elucidate ancestral pterosaur diets (Sookias, 2016). However, *Scleromochlus* and *Euparkeria* are anatomically different in many respects from pterosaurs (Nesbitt, 2011), thus limiting their utility in inferring the plesiomorphic Pterosauromorpha and Pterosauria dietary conditions.

*Scleromochlus* has been interpreted as an agile insectivore based on its elongate hind limbs (Benton, 1999). This is consistent with a hypothesis of insectivory as the ancestral pterosaur diet (Padian, 1980), although this was an extrapolation from the observation that insectivory is the most common diet in modern bats.

*Euparkeria* possess large orbits and serrated, laterally flattened teeth which have been interpreted as indicative of carnivory (Ewer, 1965; Senter, 2003). *Euparkeria* has been suggested as insectivorous during its early life‐cycle stages before becoming carnivorous as an adult (Senter, 2003). Sookias & Butler (2013), however, argued that *Euparkeria* fed on small insects and/or small tetrapods due to a lack of well‐developed jaw musculature for orally processing food items.

### (2) Basal‐most pterosaurs

A grade of early‐branching pterosaurs, referred to here as ‘basal‐most pterosaurs’, are found from the Upper Triassic to Lower Jurassic (*c*. 215–190 Ma) of the UK and Italy (Barrett *et al*., 2008). These pterosaurs exhibit 0.6–1.5 m wingspans, disproportionately large heads and heterodont, monocuspid dentitions (Fig. 1A) (Wild, 1984; Wellnhofer, 1991; Dalla Vecchia, 2003, 2013). They are largely interpreted as insectivorous and less frequently as piscivorous and carnivorous (Fig. 3).

Insectivory interpretations are based on comparative anatomy and functional morphology and associations (Fig. 3). The fang‐like, widely spaced dentitions of basal‐most pterosaurs have been suggested as suitable for catching insects. Buckland (1829) even speculated that the forelimbs of *Dimorphodon macronyx* resembled those of modern insectivorous bats. Numerous insect‐like fragments are known from the same deposits as *Dimorphodon* in the Blue Lias Formation, UK (Buckland, 1829). Functional morphological analyses include adductor muscle reconstructions of *Dimorphodon* and *Preondactylus buffarinii*, based on their quadrate–articular jaw joints. Relatively small areas for muscle attachments are consistent with low bite forces and rapid jaw closure for catching insects (Ősi, 2009, 2011).

Piscivory interpretations are based on comparative anatomy and an absence of ichnology (Fig. 3). The absence of identifiable ichnofossils has been used to suggest that these pterosaurs fished over water (Unwin, 2007). The deep, rounded snout of *Dimorphodon* has been compared with the rostrum of the piscivorous Atlantic puffin (*Fratercula arctica*) (Bakker, 1986). However, optimal *Dimorphodon* floating positions investigated using three‐dimensional (3D) digital reconstructions from ‘mathematical slices’ of specimen illustrations do not support this hypothesis (Henderson, 2010; Hone & Henderson, 2014). This analysis suggests that *Dimorphodon* would have had most of its body submerged and therefore did not spend much time foraging in water (Hone & Henderson, 2014).

Carnivory interpretations are based on comparative anatomy and functional morphology (Fig. 3). Rapid jaw closure, suggested by adductor muscle reconstructions, may have helped in the capture of small vertebrates (Ősi, 2011; Dalla Vecchia, 2013). Morphological reassessments of *Dimorphodon* forelimbs and pectoral girdle suggest an erect posture which would have facilitated terrestrial foraging for small vertebrates (Witton, 2015b).

### (3) Anurognathidae

Anurognathids are known from the Middle–Upper Jurassic (161–145 Ma) of Germany, Central Asia and China (Barrett *et al*., 2008). These pterosaurs exhibit 0.4–0.9 m wingspans (although most specimens appear to be juveniles), short box‐like skulls and large orbits (Fig. 1B) (Bakhurina & Unwin, 1995; Bennett, 2003b, 2007). Anurognathids are largely interpreted as insectivorous and less frequently as piscivorous (Fig. 3).

Insectivory interpretations are based on comparative anatomy and functional morphology, associations and (an absence of) ichnofossils (Fig. 3). *Anurognathus ammoni* possess short, pointed teeth, argued to be suitable for catching insects, and are from deposits with numerous insect fossils (Bennett, 2003b, 2007). Several anurognathids possess bristle‐like pycnofibres protruding from near their jawlines, superficially similar to bristle‐like structures seen in modern nightjars (Caprimulgidae) (Bakhurina & Unwin, 1995; Bennett, 2007). Anurognathids have thus been hypothesised as ‘aerial hawkers’; catching insects on the wing with their mouths open (Wellnhofer, 1991; Bakhurina & Unwin, 1995; Unwin, Lü & Bakhurina, 2000; Bennett, 2003b, 2007). Analyses of functional morphology include examinations of their posteriorly positioned quadrate–articular joints, indicating gape angles similar to nightjars (Bennett, 2007; Ősi, 2011; Habib & Witton, 2013). High structural strengths and bending resistances in *Anurognathus* humeri and femora signify sharp turning abilities in flight, consistent with hawking behaviour (Habib & Hall, 2012; Habib & Witton, 2013).

Piscivory interpretations are based on comparative anatomy and associations (Fig. 3). *Batrachognathus volans* and *Jeholopterus ningchengensis* are known from lacustrine deposits and exhibit slightly recurved teeth (Rjabinin, 1948; Bakhurina & Unwin, 1995; Wang *et al*., 2002), consistent with consumption of small fish and insects.

### (4) Campylognathoididae

Campylognathoidids are from Upper Triassic–Lower Jurassic (*c*. 215–176 Ma) of Central Europe and Greenland (Barrett *et al*., 2008). These pterosaurs exhibit 0.7–1.8 m wingspans and some form of heterodont and/or multi‐cusped dentition (Fig. 1C) (Wild, 1978; Padian, 2008a; Ősi, 2011; Dalla Vecchia, 2013). Campylognathoidids are most commonly interpreted as pisciviorous, with insectivory, carnivory, durophagy, herbivory/frugivory and generalism also suggested (Fig. 3).

Piscivory interpretations are based on comparative anatomy and functional morphology, content fossils and an absence of terrestrial ichnofossils (Fig. 3). The content fossil consists of a *Eudimorphodon ranzii* with scales in its stomach from pholidophorid fish (Wild, 1978; Dalla Vecchia, 2003). *Eudimorphodon* exhibits serrated, monocuspid, tricuspid and pentacuspid teeth (Wild, 1978; Stecher, 2008), which could have assisted in cutting through fish scales (Ősi, 2011). Reconstructions of *Eudimorphodon* adductor muscles suggest high quadrate mobility and rapid jaw closure when fishing (Ősi, 2011; Dalla Vecchia, 2013).

Insectivory interpretations are based on comparative anatomy and associations (Fig. 3). *Carniadactylus rosenfeldi* exhibits few wear facets on its teeth which suggests a preference for soft invertebrates (Ősi, 2011).

Carnivory interpretations are based on comparative anatomy (Fig. 3). *Eudimorphodon* and *Austriadactylus cristatus* dentitions have been interpreted as suitable for predating small vertebrates (Dalla Vecchia, 2013).

Durophagy interpretations are based on comparative anatomy (Fig. 3). Enamel spalling on *Caviramus filsurensis* teeth has been used to suggest a preference for hard crustaceans (Stecher, 2008; Dalla Vecchia, 2013).

Herbivory/frugivory interpretations are based on comparative anatomy (Fig. 3). *Carniadactylus* dentitions have also been inferred to have allowed exploitation of plant material (Ősi, 2011).

Generalism interpretations are based on comparative anatomy and functional morphology (Fig. 3). The diversity of campylognathoidid dentitions are argued to have allowed exploitation of numerous dietary items (Padian, 2008a; Witton, 2013). Functional morphological analysis subjecting 3D reconstructions (see Section IV.2) of the *Eudimorphodon* skull and dentition to dorso‐ventrally directed forces reveal high bite forces for its skull length (Henderson, 2018). This is consistent with processing diverse food items (Henderson, 2018).

### (5) Rhamphorhynchidae

#### (a) *Rhamphorhynchinae*

Rhamphorhynchines are from the Middle–Upper Jurassic of Europe and China (Unwin, 1996; Barrett *et al*., 2008). These pterosaurs exhibit wingspans up to 2 m, elongate snouts (Fig. 1D) and gracile hindlimbs (Padian, 2008b). Rhamphorhynchines are mostly interpreted as piscivorous with a few suggestions of insectivory, carnivory and durophagy (Fig. 3).

Piscivory interpretations are based on content fossils, comparative anatomy functional morphology, associations and an absence of terrestrial ichnofossils (Fig. 3). Several *Rhamphorhynchus muensteri* contain fish and nektonic invertebrate remains (Wellnhofer, 1975; Frey & Tischlinger, 2012; Hone, Habib & Lamanna, 2013; Hone *et al*., 2015a). Comparative anatomical evidence includes similarities between the conical, anteriorly pointed teeth of *Rhamphorhynchus* and those of modern gharials (*Gavialis gangeticus*), and a rhamphotheca at the anterior end of the jaw, possibly for skim‐feeding on fish (Bakker, 1986; Wellnhofer, 1991; Padian, 2008b). Prolonged skimming, however, is unlikely as flume tank experiments with *Rhamphorhynchus* jaw replicas show skimming to be energetically expensive (Humphries *et al*., 2007).

Durophagy interpretations are based on comparative anatomy (Fig. 3). Some *Dorygnathus* cf. *bathensis* teeth exhibit enamel spalling, supposedly representing damage from the consumption of hard items (Ősi, 2011).

Insectivory and carnivory interpretations are based on comparative anatomy (Fig. 3). Histological thin sections of *Rhamphorhynchus* bones from different‐sized individuals reveal that *Rhamphorhynchus* hatchlings had slow growth rates and were potentially unable to fly (Prondvai *et al*., 2012). Young *Rhamphorhynchus* were suggested to have fed on insects and small vertebrates before learning to fly, although the possibility of post‐hatching feeding by parents cannot be ruled out (Prondvai *et al*., 2012).

#### (b) *Scaphognathinae*

Scaphognathines are from the Middle–Upper Jurassic of Europe, Asia and Cuba (Barrett *et al*., 2008). These pterosaurs exhibit 0.7–2.5 m wingspans and stout skulls (Fig. 1E) (Witton, 2013). Piscivory is the most common interpreted diet, with insectivory, carnivory and generalism also suggested (Fig. 3).

Piscivory interpretations are based on content fossils, comparative anatomy, functional morphology and an absence of terrestrial ichnofossils (Fig. 3). A *Scaphognathus crassirostris* specimen was described with fish remains in its throat and mouth, corroborating earlier morphological comparisons likening *Scaphogathus* teeth to gharials (Seeley, 1901; Stieler, 1922; Bennett, 2014). Functional morphological evidence comes from flight models suggesting that *Scaphognathus* exhibited similar soaring profiles to modern gulls (Laridae) and albatrosses (Diomedeidae) (Rayner, 1989; Witton, 2008).

Insectivory interpretations are based on associations and comparative anatomy (Fig. 3). Lü & Bo (2011) hypothesised *Jianchangopterus zhaoianus* from the Tiaojishan Formation, China, as an obligate insectivore as a result of strict niche partitioning with unrelated, potentially piscivorous, pterosaurs.

Carnivory interpretations are based on association (Fig. 3). *Scaphognathus* has been depicted predating *Anurognathus* (Bakker, 1986).

Generalism interpretations are based on comparative anatomy (Fig. 3). Witton (2013) reasoned their relatively robust snouts and claws enabled terrestrial foraging.

### (6) Basal monofenestratans

Basal monofenestratans are from the Middle–Upper Jurassic (165–151 Ma) of China (Lü *et al*., 2010) and the UK (Martill & Etches, 2013; Witton, O'Sullivan & Martill, 2015). These pterosaurs exhibit 0.8–1.2 m wingspans with elongated heads and necks like pterodactyloids (Fig. 1F), and short bodies and extended tails like non‐pterodactyloids (Lü *et al*., 2010; Wang *et al*., 2010a; Witton *et al*., 2015). Basal monofenestratans are interpreted as insectivorous, piscivorous and carnivorous (Fig. 3).

Insectivory interpretations are based on associations and comparative anatomy (Fig. 3). Chinese basal monofenestratans are from terrestrial deposits of the Tiaojishan Formation and Daohugou Bed, which contain numerous insects (Lü *et al*., 2010, 2011b). Different dentitions among basal monofenestratans have been interpreted as indicative of strict niche partitioning (Lü *et al*., 2010, 2011b). *Darwinopterus robustadens* for example exhibits relatively stout teeth, perhaps for consuming insects with thicker exoskeletons (Lü *et al*., 2010, 2011b).

Piscivory interpretations are based on associations and comparative anatomy (Fig. 3). The Tiaojishan Formation and Daohugou Bed also contain numerous fish fossils, thus Chinese basal monofenestratans have been associated with facultative piscivory (Wang *et al*., 2010b). European species such as *Cuspicephalus scarfi* from coastal deposits possess high tooth counts (Martill & Etches, 2013), potentially for grabbing fish (Witton *et al*., 2015).

Carnivory interpretations are based on associations and comparative anatomy (Fig. 3). Initial descriptions of *Darwinopterus modularis* noted spike‐like teeth for gripping vertebrate prey, such as gliding mammals and other pterosaurs, which are known from Tiaojishan (Lü *et al*., 2010).

### (7) Istiodactylidae

Istiodactylids are from the Lower Cretaceous (130–112 Ma) of the UK and China (Barrett *et al*., 2008) with 2.5–4.5 m wingspans, rounded anterior rostra and labiolingually flattened, interlocking teeth in the anterior halves of their jaws (Fig. 1G) (Witton, 2013). Istiodactylids are interpreted as piscivorous and carnivorous, with a few suggestions of insectivory (Fig. 3).

Piscivory interpretations are based on comparative anatomy and associations (Fig. 3). Associations include numerous fish fossils from the Jiufotang Formation, where istiodactylids including *Liaoxipterus brachyognathus* and *Nurhachius ignaciobrioti* are also known (Wang & Lü, 2001; Wang & Zhou, 2006). Original reconstructions of the *Istiodactylus latidens* skull suggested that this pterosaur caught fish with its interlocking teeth (Hooley, 1913).

Carnivory interpretations are based on comparative anatomy and ichnology (Fig. 3). Ichnological evidence is from pterosaur track‐ways, used to infer ground‐based scavenging (Unwin, 2007). Howse, Milner & Martill (2001) argued that the razor‐edged teeth of *Istiodactylus* were suited for pulling and twisting off pieces of flesh. Re‐examinations of *Istiodactylus* found features indicating both mechanical strength and weakness, typical of obligate scavengers (Witton, 2012, 2013; Martill, 2014).

Insectivory interpretations are based on comparative anatomy of a *Liaoxipterus* specimen with an elongated hyoid apparatus (collection of small bones which attach to the posterior of the tongue). The proportional lengths of these bones are reportedly similar to modern chameleons (Chameleonidae), and *Liaoxipterus* was thus reasoned to have caught insects with the aid of a projectile tongue (Lü, Xu & Ji, 2008b; Lü, 2015).

### (8) Ornithocheiridae

Ornithocheirids are from Lower–Upper Cretaceous (140–95 Ma) deposits on all continents except Antarctica (Barrett *et al*., 2008). These pterosaurs had 4–8 m wingspans and elongated jaws with large teeth at the anterior end and smaller teeth towards the posterior end (Fig. 1H) (Ősi, 2011; Witton, 2013; Elgin, 2014). Ornithocheirids are mostly interpreted as piscivorous, with a few suggestions of filter‐feeding (Fig. 3).

Piscivory interpretations are based on content fossils, associations, ichnology, isotope analyses, comparative anatomy and functional morphology (Fig. 3). Most ornithocheirids are known from lagoonal, coastal and marine deposits (Frey, Martill & Buchy, 2003; Chatterjee & Templin, 2004; Unwin, 2006; Molnar & Thulborn, 2007; Unwin & Martill, 2007; Kear, Deacon & Siverson, 2010; Veldmeijer *et al*., 2012; Wretman & Kear, 2013). A coprolite interpreted to be from *Guidraco venator* contains fish bones (Wang *et al*., 2012). Carbon isotope ratios of ornithocheirid teeth indicate consumption of freshwater and shallow marine fish (Amiot *et al*., 2010). These teeth however contained high proportions of dentine which can easily be altered through diagenesis (Amiot *et al*., 2010). Analysis of teeth with distinctive enamel alternatively found carbon ratios similar to those typical of marine environments (Tütken & Hone, 2010). Most ornithocheirids such as *Ornithocheirus mesembrinus* possess long conical teeth, supposedly for grasping fish (Wellnhofer, 1991; Dalla Vecchia, 1993; Fletcher & Salisbury, 2010; Ősi, 2011; Veldmeijer *et al*., 2012; Wang *et al*., 2014a). Functional modelling of energy costs using body mass estimates indicate that catching fish whilst on the wing and from water surfaces incurred low energetic costs and would have been energy efficient for *Anhanguera piscator* (Habib, 2015).

Filter‐feeding interpretations are based on comparative anatomy (Fig. 3). *Boreopterus cuiae* and *Zhenyuangopterus longirostris* possess slender, elongated teeth, interpreted to have trapped small aquatic organisms before straining and swallowing (Wang & Zhou, 2006; Witton, 2013; Teng *et al*., 2014).

### (9) Pteranodontoidea

#### (a) *Pteranodontidae*

Pteranodontids are from the Lower–Upper Cretaceous (100–80 Ma) of North America and Europe (Barrett *et al*., 2008). These pterosaurs had wingspans up to 6.5 m, narrow edentulous jaws (Fig. 1I) and exhibit sexual dimorphism, with larger head crests in supposed males (Bennett, 2001). Pteranodontids are commonly interpreted as piscivorous, with a few suggestions of durophagy (Fig. 3).

Piscivory interpretations are based on content fossils, associations, comparative anatomy and functional morphology (Fig. 3). Content fossils include fish remains within several *Pteranodon* stomachs (Brown, 1943; Hargrave, 2007). Pteranodontids are exclusively known from marine deposits and have thus been suggested as albatross analogues (Marsh, 1876; Eaton, 1910; Wellnhofer, 1991; Bennett, 1993, 1994; Unwin, 2006; Witton, 2013). Comparative anatomical analyses agree on piscivory (Fig. 3). Skim‐feeding was initially proposed as *Pteranodon* and modern avian skimmers (*Rynchops* spp.) possess similarly narrow jaws (Marsh, 1876; Eaton, 1910; Zusi, 1962). Spiral‐shaped joints between the quadrate and articular in the lower jaw were interpreted as attachment points for throat sacs for scooping up fish (Eaton, 1910; Hankin, 1912). However, cervical vertebrae of *Pteranodon* were later judged too small for scooping and skimming for fish (Witton, 2013). Functional morphological analyses have tested the feasibility of feeding behaviours. Bramwell & Whitfield (1974) placed scaled replicas of the *Pteranodon* skeleton in wind‐tunnels and inferred slow flight and gliding speeds to help catch fish whilst on the wing. By contrast, other early flight models found *Pteranodon* flight profiles were more suited for skim‐feeding, although their body masses were extrapolated from modern seabirds (Hazlehurst & Rayner, 1992). Later energy expenditure modelling found that capturing aquatic organisms on the wing or whilst resting on water surfaces were energetically feasible behaviours (Habib, 2015).

Durophagy interpretations are based on content fossils (Fig. 3); a few (disputed) crustacean remains have been found within *Pteranodon* throats (Brown, 1943; Bennett, 2001).

#### (b) *Nyctosauridae*

Nyctosaurids are known from the Upper Cretaceous (89–66 Ma) of Mid‐West USA, Mexico and Brazil (Barrett *et al*., 2008). These pterosaurs exhibit 2–3 m wingspans and gracile morphologies, with *Nyctosaurus gracilis* exhibiting an ‘antler‐like’ head crest (Fig. 1J) (Bennett, 2003a). Nyctosaurids are exclusively interpreted as piscivorous (Fig. 3), based on associations, comparative anatomy, functional morphology and ichnology (Fig. 3). All nyctosaurids are known from shallow marine deposits (Marsh, 1876; Bennett, 2003a; Frey *et al*., 2006) with an absence of ichnofossils suggesting foraging over water (Unwin, 2007). Comparative anatomy and functional morphology of mineralised wing tendons in *Muzquizopteryx coahuilensis* allowed theoretical wing muscle reconstructions indicating restricted wing movements and foraging in shallow marine environments (Frey *et al*., 2006).

### (10) Ctenochasmatoidea

#### (a) *Basal ctenochasmatoids*

Basal ctenochasmatoids are from the Upper Jurassic (157–145 Ma) of Germany (Barrett *et al*., 2008). Basal ctenochasmatoids have different morphologies; *Pterodactylus* species exhibit straight, thin jaws with around 50–70 small, conical teeth (Fig. 1K) (Arthaber, 1921; Bennett, 2012); *Cycnorhamphus suevicus*, by contrast, exhibits a bizarre arrangement where the anterior portions of the upper and lower jaws curve downward and upward, respectively, with rounded teeth (Witton, 2013). Basal ctenochasmatoids are commonly interpreted as insectivorous and piscivorous with single suggestions of carnivory, durophagy, filter‐feeding and generalism (Fig. 3).

Insectivory interpretations are based on comparative anatomy and ichnology (Fig. 3). Contemporaneous ichnofossils suggest that basal ctenochasmatoids foraged for insects on the ground (Unwin, 2007). *Pterodactylus* was originally suggested to have caught flying insects because their jaws superficially resemble the beaks of modern bee‐eaters (Meropidae) (Arthaber, 1921; Bennett, 2012).

Piscivory interpretations are based on comparative anatomy and associations (Fig. 3). *Pterodactylus* dentitions have alternatively been suggested to be adapted for snatching small fishes from water surfaces (Rayner, 1989; Bennett, 2012).

Carnivory interpretations are based on ichnology (Fig. 3). Contemporaneous ichnofossils suggest that basal ctenochasmatoids hunted and/or scavenged for vertebrates on the ground (Unwin, 2007).

Durophagy interpretations are based on comparative anatomy (Fig. 3). The *Cycnorhamphus* jaw and dentition has been interpreted as an adaptation for crushing crustacean exoskeletons (Witton, 2013).

Filter‐feeding interpretations are based on comparative anatomy (Fig. 3). The unspecialised teeth of basal ctenochasmatoids have been suggested to have facilitated basic filter‐feeding in shallow water bodies (Unwin, 2006).

Generalism interpretations are based on functional morphology (Fig. 3). A 3D model of the skull subjected to dorso‐anteriorly directed forces found that *Pterodactylus* had intermediate bite forces and thus perhaps consumed diverse food items (Henderson, 2018).

#### (b) *Ctenochasmatidae*

Ctenochasmatids are from the Lower–Upper Cretaceous (152–100 Ma) of Argentina, Central and Western Europe, Morocco, and East Asia (Barrett *et al*., 2008). These pterosaurs exhibit 1–2.5 m wingspans and elongated rostrums with densely spaced slender teeth of assorted sizes (Fig. 1L). Ctenochasmatids are mostly interpreted as filter‐feeders with few cases of piscivory and durophagy (Fig. 3).

Filter‐feeding interpretations are based on comparative anatomy, content fossils, ichnofossils and functional morphology (Fig. 3). Content fossils include *Pterodaustro guinazui* specimens from the Largarcitio Formation, Argentina with gravel‐sized gastroliths in their stomachs, potentially for crushing planktonic‐sized organisms (Codorniú, Chiappe & Cid, 2013). Contemporaneous ichnofossils suggest that ctenochasmatids foraged in shallow water bodies (Unwin, 2007). Comparative anatomical interpretations are based on their highly derived jaws and dentitions (Chiappe & Chinsamy, 1996; Chiappe *et al*., 1998; Naish & Martill, 2003; Chinsamy‐Turan, Codorniú & Chiappe, 2008). *Ctenochasma gracile*, for example, possesses straight jaws with around 260 teeth, and *Gnathosaurus subulatus* possesses around 130 teeth in jaws that end in a disc‐like structure similar to modern spoonbills (Plataleinae) (Fig. 1L) (Howse & Milner, 1995). Functional morphological analysis of 3D models of *Ctenochasma*, *Gnathosaurus* and *Pterodaustro* skulls subjected to dorso‐anteriorly directed forces found that ctenochasmatids had exceptionally weak bites and could thus only feed on planktonic‐sized food items (Henderson, 2018).

Piscivory interpretations are based on comparative anatomy (Fig. 3). Sharp, slender teeth are inferred to have aided in catching fish (Lü, Kundrat & Shen, 2016a).

Durophagy interpretations are based on content fossils and comparative anatomy (Fig. 3). *Pterodaustro* exhibit short, rounded teeth in their upper jaws, perhaps for crushing hard‐shelled crustaceans (Chinsamy, Codorniú & Chiappe, 2009; Codorniú *et al*., 2013).

### (11) Lonchodectidae

Lonchodectids are poorly known pterosaurs known from the Lower–Upper Cretaceous (140–90 Ma) of China, the UK and Brazil (Unwin, 1996, 2006; Unwin, Wang & Meng, 2008). These pterosaurs possess elongated jaws with either slightly dorso‐ventrally flattened tooth crowns, or slender recurved teeth (Fig. 1M). Lonchodectids are mostly interpreted as generalists, along with piscivory and carnivory (Fig. 3).

Generalism interpretations are based on comparative anatomy and associations (Fig. 3). *Lonchodectes* is known from the shallow marine Cambridge Greensand Formation of the UK, which is suggested to have contained numerous aquatic organisms which could have supported generalist diets (Unwin, 1996, 2006). The dorso‐ventrally flattened teeth of *Lonchodectes* were reasoned to have facilitated the handling and consumption of varied food items (Unwin, 1996, 2006).

Piscivory interpretations are based on comparative anatomy and an absence of terrestrial ichnofossils (Fig. 3). Slender, recurved teeth in the anterior halves of their jaws (Fig. 1M) have been argued to be suitable for catching fish (Wang & Zhou, 2006; Lü *et al*., 2016b).

Carnivory interpretations are based on comparative anatomy (Fig. 3). *Feilongus* dentitions have been interpreted as suitable for predatory lifestyles (Wang *et al*., 2014b).

### (12) Dsungaripteroidea

#### (a) *Basal dsungaripteroids*

Basal dsungaripteroids are known from the Upper Jurassic (157–145 Ma) of Central and Western Europe, USA and Tanzania (Barrett *et al*., 2008). These pterosaurs exhibit 1 m wingspans and small, dorsally positioned sagittal crests on their skulls (Fig. 1N) (Wellnhofer, 1991; Witton, 2013). Basal dsungaripteroids are exclusively interpreted as durophagous, based on comparative anatomy (Fig. 3). *Germanodactylus* for example, exhibits edentulous jaw tips and low‐crowned teeth, supposedly for selecting and crushing bivalve and crustacean shells (Bennett, 2006; Unwin, 2006).

#### (b) *Dsungaripteridae*

Dsungaripterids are from the Lower Cretaceous (145–100 Ma) of China, Mongolia and South America (Barrett *et al*., 2008; Dececchi *et al*., 2014). These pterosaurs exhibit 2–5 m wingspans, relatively robust skeletons and laterally flattened, edentulous jaw anteriors (Fig. 1O) (Young, 1964; Lü *et al*., 2009; Hone *et al*., 2015b). Dsungaripterids are commonly interpreted as durophagous with some suggestions of piscivory (Fig. 3).

Durophagy interpretations are based on comparative anatomy, ichnofossils, associations and functional morphology (Fig. 3). Contemporaneous ichnofossils suggest that dsungaripterids foraged for hard‐shelled organisms in shallow water bodies (Unwin, 2007). Associations indicate that dsungaripterids are mostly found in inland fluviolacustrine deposits (Matsukawa *et al*., 2006). Comparative anatomical evidence derives from jaws and teeth. *Dsungaripterus weii*, for example, exhibits upturned jaw tips with low‐crowned, anvil‐shaped teeth in the posterior half of its jaws (Young, 1964; Wellnhofer, 1991), suggesting *Dsungaripterus* might have picked out bivalves, gastropods and crabs with its jaw tips before cracking open their shells or exoskeletons with its teeth (Young, 1964; Wellnhofer, 1991; Lü *et al*., 2009). Functional morphology of 3D models of *Dsungaripterus* reveals awkward floating positions (see Section IV.2), indicative of a pterosaur better adapted for foraging in shallow waters (Hone & Henderson, 2014).

Piscivory interpretations are based on comparative anatomy (Fig. 3). The straight jaw tips and relatively slender, conical teeth of *Noripterus complicidens* and *‘Phobetor’ parvus* were interpreted as suitable for catching fish (Wellnhofer, 1991; Lü *et al*., 2009; Veldmeijer *et al*., 2012).

### (13) Tapejaridae

Tapejarids are from the Lower–Upper Cretaceous (130–93 Ma) of Brazil, Spain and China (Barrett *et al*., 2008; Vullo *et al*., 2012). These pterosaurs had 1.5–3 m wingspans, relatively elongated hindlimbs and sail‐like cranial crests (Fig. 1P) (Wang & Zhou, 2006; Witton, 2013). Many different diets are interpreted for tapejarids, including herbivory/frugivory, durophagy, carnivory, piscivory and insectivory (Fig. 3).

Herbivory/frugivory interpretations are based on comparative anatomy, functional morphology and associations (Fig. 3). *Tapejara wellnhoferi* possess edentulous, anteroventrally curved jaws (Fig. 1P) which were likened to modern frugivorous parrots (Psittaciformes) (Wellnhofer & Kellner, 1991; Unwin, 2006). Other anatomical features supporting frugivory include large head crests for moving vegetation and robust phalanges and claws for moving along tree branches (Wellnhofer & Kellner, 1991; Veldmeijer *et al*., 2012). Associations are based on tapejarid occurrences correlating spatially and temporally with the emergence and spread of fruiting angiosperms (Unwin, 2006; Meijer *et al*., 2007; Wang *et al*., 2008; Vullo *et al*., 2012). Functional morphological analyses include *Tapejara* bite force estimations from the relative positions of their jugal and quadrate bones, suggesting tapejarids could readily pick up and flatten fruits with their jaws (Meijer *et al*., 2007).

Durophagy interpretations are based on associations, comparative anatomy and functional morphology (Fig. 3). The fossil records of tapejarids, seeds and gymnosperm cones in the Lower Cretaceous partially correlate (Pinheiro, Liparini & Schultz, 2014). *Tapejara* adductor muscle reconstructions show well‐developed systems for potentially consuming harder items (Pinheiro *et al*., 2014). This is corroborated by 3D constructs of the *Tapejara* skull which exhibited high bite forces for potentially cracking open seeds (Henderson, 2018).

Carnivory interpretations are based on comparative anatomy and content fossils (Fig. 3). A fossilised regurgitate pellet, tentatively identified as tapejarid in origin, contains small bird remains (Veldmeijer *et al*., 2012). Comparative anatomy includes a partial dentary from the La Huerguina Formation, Spain, which may have had a ‘cutting‐edge’ rhamphotheca for catching small vertebrates (Vullo *et al*., 2012).

Piscivory interpretations are based on associations (Fig. 3). Brazilian tapejarids are known from lagoonal and marine deposits and are reasoned to have fed on contemporaneous fish (Unwin & Martill, 2007).

Insectivory interpretations are based on comparative anatomy and associations (Fig. 3). *Nemicolopterus crypicus,* from the Jiufotang Formation, China was suggested to have pursued insects along tree branches aided by curved phalanges (Wang *et al*., 2008).

### (14) Chaoyangopteridae

Chaoyangopterids are from the Lower Cretaceous (130–112 Ma) of China and Brazil, and had 1–4 m wingspans and ‘scissor‐like’ edentulous jaws (Fig. 1Q) (Unwin & Martill, 2007; Barrett *et al*., 2008). Chaoyangopterids are interpreted as piscivorous and generalists (Fig. 3).

Piscivory interpretations are based on associations and comparative anatomy (Fig. 3). *Chaoyangopterus zhangi* is known from lacustrine deposits with numerous fishes (Wang & Zhou, 2006). Comparative anatomical evidence is based on the scissor‐like edentulous jaws of these pterosaurs helping to catch fish when on the wing (Wang & Zhou, 2006).

Generalism interpretations are based on comparative anatomy (Fig. 3). Chaoyangopterids exhibit similar limb proportions to related groups such as azhdarchids that have been proposed as ground‐based foragers (see Section IV.16) (Witton, 2013).

### (15) Thalassodromidae

Thalassodromids are from the Lower Cretaceous of Brazil (125–100 Ma) (Barrett *et al*., 2008). These pterosaurs had 2–5 m wingspans, edentulous jaws and large laterally flattened cranial crests (Fig. 1R) (Kellner & Campos, 2002b; Humphries *et al*., 2007). Thalassodromids are interpreted as piscivorous and as generalists (Fig. 3).

Piscivory interpretations are based on comparative anatomy and functional morphology (Fig. 3). *Thalassodromeus sethi* lower jaws were proposed to be similar to skimming *Rynchops* (Kellner & Campos, 2002a,*b*). Lower jaw replicas of *Thalassodromeus* and *Tupuxuara cristata* placed in flume tanks however, experienced levels of drag that would not have allowed stable skimming (Humphries *et al*., 2007).

Generalism interpretations are based on comparative anatomy (Fig. 3). Thalassodromid hind‐limbs are apparently robust, and the group was interpreted as terrestrially foraging opportunists (Witton, 2013).

### (16) Azhdarchidae

Azhdarchids are from the Upper Cretaceous (99–66 Ma) of North America, North Africa, Eastern Europe and Asia (Barrett *et al*., 2008; Averianov *et al*., 2015). These pterosaurs exhibit 1.5–11 m wingspans and possess elongated necks and skulls (Fig. 1S), and disproportionally small bodies and feet for their size (Paul, 1987; Witton & Naish, 2008). Azhdarchids are interpreted as carnivorous and piscivorous with some suggestions of generalism, durophagy, insectivory and herbivory/frugivory (Fig. 3).

Carnivory interpretations are based on comparative anatomy, associations, ichnology and functional morphology (Fig. 3). Comparative anatomical interpretations include long necks and jaws (Fig. 1S) for carcass probing (Lawson, 1975; Wilkinson & Ruxton, 2012) and/or aerial predation (Nessov, 1984; Chatterjee & Templin, 2004). Their necks, however, have been reinterpreted as too stiff for these roles, and azhdarchids have alternatively been suggested as ground‐based predators and scavengers based on their long limbs (Witton & Naish, 2008, 2015; Witton, 2013; Naish & Witton, 2017). Where multiple azhdarchids are known, niche partitioning is suggested (Witton & Naish, 2008; Naish *et al*., 2015). The Haţeg Basin, Romania, for example contains *Hatzegopteryx thambema* with a 10 m wingspan (Witton & Naish, 2015), *Eurazhdarcho langendorfensis* with a 3 m wingspan, and an unnamed azhdarchid with a 3 m wingspan and short, wide cervical vertebrae (Vremir *et al*., 2015). *Hatzegopteryx* was reasoned to have consumed the largest prey, with the short‐necked azhdarchid consuming larger prey than *Eurazhdarcho* as its neck potentially offered greater mechanical advantages (Vremir *et al*., 2015).

Piscivory interpretations are based on comparative anatomy, functional morphology and ichnofossils (Fig. 3). Possible azhdarchid track‐ways on mudflats suggest wading behaviours (Unwin, 2007). However azhdarchid feet have been argued to have been too small to have provided support on sandy, muddy ground (Witton & Naish, 2008). Comparative anatomy of azhdarchid necks suggests some articulation at their shoulders which may have allowed their heads rudimentarily to bend forward and seize fish on the wing (Martill *et al*., 1998; Chatterjee & Templin, 2004; Averianov, 2013). Functional morphology indicates azhdarchid gapes similar to *Rynchops,* based on jaw bone articulations, suggesting skim‐feeding (Kellner & Langston, 1996; Ősi, 2004). However this was not supported by flume tank results indicating that skim‐feeding was energetically unfeasible (Humphries *et al*., 2007). Theoretical reconstructions of possible azhdarchid throat pouches suggest that scooping would have put incredibly high strain on their necks (Witton & Naish, 2015).

Durophagy interpretations are based on comparative anatomy and associations (Fig. 3). Contemporaneous invertebrate burrows led to the suggestion that azhdarchids were sediment probers for hard‐shelled organisms (Wellnhofer, 1991). Jaw fragments from Morocco possess bony protuberances tentatively interpreted as structures for crushing mollusc shells (Martill & Ibrahim, 2015).

Generalism interpretations are based on comparative anatomy and associations (Fig. 3). Numerous azhdarchid remains from terrestrial deposits led to the hypothesis that azhdarchids were opportunistic ground‐based foragers, with larger genera consuming more animals for higher energy returns (Witton & Naish, 2008, 2015; Witton, 2013).

Insectivory interpretations are based on ichnology (Fig. 3). Azhdarchid tracks on mudflats from the Unhangari Formation, South Korea, were interpreted as indicative of foraging for insects (Hwang *et al*., 2002).

Herbivory/frugivory interpretations are based on comparative anatomy (Fig. 3). Azhdarchids have been reasoned to have fed on fruits given some jaw similarities to tapejarids (see Section IV.14) (Ősi, Weishampel & Jianu, 2005).

## V. DISCUSSION

### (1) Why is there a lack of consensus in pterosaur diet research?

Overall, there is limited consensus on diets for most pterosaur groups. Most dietary interpretations are supported by qualitative evidence, most commonly from comparative anatomy. There is strong consensus on diets for some pterosaur groups, such as insectivory in Anurognathidae and piscivory in Ornithocheiridae and Pteranodontidae, supported by one or several evidential categories. For other groups there is little consensus: in both basal ctenochasmatoids and Azhdarchidae, for example, six distinct diets have been suggested. In general, pterosaur principal groups with more species exhibit a higher diversity of dietary hypotheses. Choice of phylogeny has some impact on consensus, with the largest differences observed between phylogenies for non‐monofenestratan groups. Numbers of dietary interpretations exhibit small increases for much of pterosaur research history, with most interpretations proposed in the 21st century. Changes in the number of dietary interpretations through time do not correlate with numbers of pterosaur publications.

That the majority of dietary interpretations are qualitative is unsurprising because such interpretations can be readily proposed without rigorous experiments or analyses. Comparative anatomy uses extrapolations from observational, and occasionally experimental, studies of extant organisms (Aerts, 1990; Kellner & Campos, 2002b; Witton & Naish, 2008). Associations similarly rely on straightforward understanding of modern food webs when reconstructing ecological relationships between contemporaneous taxa, and of geological processes when interpreting depositional environments of specimens (Wellnhofer, 1991; Frey & Tischlinger, 2012). Content fossils are often interpreted as direct records of trophic interactions between extinct organisms (Hone & Faulkes, 2014; Hone *et al*., 2015a; Witton, 2018), although their rarity limits their utility, and there is an inbuilt bias towards preservation of food items consumed immediately prior to death, which may be atypical (Davis & Pineda‐Munoz, 2016). Furthermore, the usefulness of gut contents for indicating typical diet in animals that consume a range of items is limited when small sample sizes are all that is available. Analyses based on extant predators indicate that large numbers of individuals, from tens to even hundreds, need to be sampled to capture a true picture of diet (e.g. Szczepanski & Bengtson, 2014). Ichnofossils are more common, although the poor quality and uncertainty regarding the makers of many specimens limits robust ecological interpretations (Lockley & Wright, 2003; Lockley, Harris & Mitchell, 2008).

Many quantitative methods were not developed and readily utilised until the late 20th and early 21st centuries (Aerts, 1990; Lauder, 1995; Rayfield *et al*., 2001), and are more difficult to use because: (*i*) they often require specialist technology and/or equipment; (*ii*) they require thorough understanding of experimental design and hypothesis testing; (*iii*) they can require destructive sampling of specimens (Aerts, 1990; Anderson *et al*., 2011). Robust quantitative methods have therefore been unavailable for much of pterosaur research history.

There are several reasons why different groups of pterosaurs exhibit different levels of consensus regarding dietary interpretations. The observed correlation between species and numbers of dietary interpretations per principal group provides one possible explanation. Pterosaur groups that exploited more new food sources in their respective palaeoenvironments potentially underwent greater eco‐morphological changes which facilitated further exploitation, likely resulting in more speciation events (Zhou *et al*., 2017). Zhou *et al*. (2017) explored this idea by morphologically quantifying pterosaur skulls, jaws and dentitions with respect to assigned diets from the literature. The regions of morphospace occupied by pterosaurs were indeed better explained by their respective assigned diet than by evolutionary relatedness (Zhou *et al*., 2017). Dietary assignments by Zhou *et al*. (2017), however, were primarily based on qualitative evidence and/or untested hypotheses from the literature and should therefore be interpreted with caution. The disparity in levels of consensus regarding diet across Pterosauria as shown in the current study illustrates the difficulty in reliably uncovering biological signals that may explain pterosaur dietary diversity.

Furthermore, pterosaur diets are unlikely to have fallen into discrete categories as described herein and in Zhou *et al*. (2017). Many interpretations of pterosaur feeding behaviours and diets from morphological evidence concern only what they appear to be optimally adapted to eat. This is because pterosaur functional attributes are mostly inferred from comparisons of skeletal features with analogous structures in modern taxa, under the assumption that similarity in morphology correlates with similarity in function (Lauder, 1995). However, other pterosaur tissues (muscles, nervous system, etc.) are rarely preserved and pterosaur structures may have had other functions related or unrelated to feeding, and flexibility in feeding behaviours that cannot be inferred from hard tissues alone (Lauder, 1995). This compromises the assumption that pterosaurs were adapted to consume only one type of food. Furthermore, pterosaurs might have occasionally consumed food items outside of their normal dietary ranges (see online Appendix S1). A modern example involves fruits and seeds found in the stomachs of several crocodilian species (Platt *et al*., 2013a). Crocodilians are renowned predators, but fruits and seeds have been found in high enough quantities to rule out accidental consumption [see Platt *et al*. (2013a) and references therein]. Although incorporating potential dietary plasticity would be very useful for palaeoecosystem reconstructions, we currently do not have the techniques to do so.

The lack of consensus in pterosaur dietary interpretations is more likely explained by non‐biological and historical signals. The patchy quality of the pterosaur fossil record (Butler *et al*., 2012, 2013; Dean *et al*., 2016) is one non‐biological example as some dietary interpretations are made on few and/or poorly preserved specimens, resulting in low confidence levels. Buckland (1829), for example, hypothesised insectivory in *Dimorphodon* based on the limited post‐cranial material known at the time. Only with the description of the skull decades later was piscivory hypothesised (Seeley, 1901). Lonchodectids are interpreted as piscivores and generalists, but are mostly known from fragmentary remains (Unwin, 1996; Lü *et al*., 2016b). Basal dsungaripteroids are universally interpreted as durophagous but few dietary interpretations have been proposed, at least in part because their fossil record is poor (Bennett, 2006; Barrett *et al*., 2008). Pterosaurs from Lagerstätten and other deposits with well‐preserved remains generally have higher levels of dietary agreement. Pteranodontids are known from huge specimen numbers from the Niobrara Formation (Marsh, 1876; Eaton, 1910; Brown, 1943). Anuroganthids are known from few, but extremely well preserved, specimens from the Solnhofen Limestone and Tiaojishan and Yixian formations (Rjabinin, 1948; Bennett, 2003b, 2007). These finds have allowed thorough investigations into the functional morphology of these pterosaurs to test interpretations of piscivory and insectivory respectively (Bramwell & Whitfield, 1974; Hazlehurst & Rayner, 1992; Habib & Hall, 2012; Habib & Witton, 2013; Habib, 2015). This highlights the importance of finding more, and ideally well‐preserved, specimens to generate and test dietary hypotheses.

Of all pterosaurs, azhdarchids have the greatest diversity of dietary interpretations, yet they are known from relatively fragmentary remains (Lawson, 1975; Cai & Wei, 1994; Vremir *et al*., 2015). Azhdarchids are the largest flying organisms ever to have lived, thus it is reasonable to suggest that these pterosaurs have received disproportionate levels of scientific study (e.g. Lawson, 1975; Wellnhofer, 1991; Cai & Wei, 1994; Martill, 1997; Martill *et al*., 1998; Hwang *et al*., 2002; Chatterjee & Templin, 2004; Witton & Naish, 2008, 2015; Brown, 2015; Martill & Ibrahim, 2015; Vremir *et al*., 2015). Higher levels of study on specimens of limited number and quality increase the likelihood of tentative, yet varied, dietary interpretations. This further indicates how levels of dietary consensus are at least partially confounded by non‐biological signals.

Disputes over evolutionary relationships also mask biological signals of pterosaur diets. Placements of species within different groups make it difficult to elucidate whether dietary ranges are representative for principal groups or an artefact of the chosen phylogeny. This is especially problematic for non‐monofenestratan groups because they exhibit lower morphological disparity than monofenestratan pterosaurs (Prentice *et al*., 2011; Zhou *et al*., 2017). The Andres phylogeny is an example of this as it contains fewer, more inclusive non‐monofenestratan groups than the other phylogenies. Better resolution of the membership of pterosaur principal groups would therefore assist with elucidating true dietary ranges for each group. Better specimens of known taxa would assist with more confident phylogenetic placements.

The absence of correlation between numbers of pterosaur publications and dietary interpretations through time is unsurprising. Through the 19th and early 20th centuries, pterosaur publications mainly comprised species descriptions and systematics from European and North American Lagerstätten such as Solnhofen and the Niobrara Chalk (Marsh, 1876; Seeley, 1901; Eaton, 1910; Arthaber, 1921). Very few of these studies hypothesised diets as during this time period ecology was a largely unrecognised area of scientific study. The slight decrease in publications during the middle 20th century saw few new specimen and species descriptions (Rjabinin, 1948). The increase in publications in the latter half of the 20th century coincided with discoveries of new Lagerstätten, including the Santana and Crato formations in Brazil (Wellnhofer & Kellner, 1991; Unwin & Martill, 2007), and the Yixian Formation in China (Ji, Ji & Padian, 1999), as well as a broader renaissance in reptile palaeobiology. New, morphologically distinct, pterosaur groups were discovered during this time, resulting in new hypothesised diets such as filter‐feeding in ctenochasmatids (Bakker, 1986), herbivory/frugivory in tapejarids (Wellnhofer & Kellner, 1991) and generalism in lonchodectids (Unwin, 1996). The last quarter of the 20th century also saw palaeontological research expand from mostly description‐ and systematic‐based studies to focus also on the wider biology and ecology of extinct taxa (Bramwell & Whitfield, 1974; Bramble, 1978; Bakker, 1986; Witmer, 1995). Many pterosaurs described during and after this time included some ecological interpretation, including possible diet, but many of these interpretations were constructed from simple qualitative comparisons and analogies with modern biology. The 1 year in which numbers of dietary interpretations exceeded that of pterosaur publications, 1991, can be explained by the publication of a comprehensive summary and interpretation of pterosaur research up to that time, including dietary interpretations for most pterosaur groups (Wellnhofer, 1991) (see online Appendix S1). Further pterosaur specimen discoveries, e.g. the Tiaojishan and Jiufotang Formations (Wang & Zhou, 2006; Lü *et al*., 2010; Lü & Bo, 2011), continued the increase in publications into the 21st century.

Increases in the number and rate of publication and dietary interpretations is also explained in part by the application of new techniques and new types of evidence. These include biomechanical analyses (Bramwell & Whitfield, 1974; Fastnacht, 2005; Hone & Henderson, 2014; Henderson, 2018), and stable isotope analyses (Amiot *et al*., 2010; Tütken & Hone, 2010). Ichnofossil evidence was not used to inform dietary interpretations until the 21st century due to debates over whether the creators of ichnofossils were pterosaurian or crocodilian (Lockley & Wright, 2003).

Greater appreciation for pterosaurs, and extinct taxa in general, as organisms with independent evolutionary histories which faced unique selection pressures in their respective palaeoenvironments, are helping with the construction of testable hypotheses (Witton & Habib, 2010; Fiorillo *et al*., 2015). Many pterosaur dietary interpretations in the 19th and 20th centuries were based on simple extrapolations from modern flying vertebrates or semi‐aquatic reptiles (Seeley, 1901; Padian, 1980, 1983; Wellnhofer, 1991; Unwin & Henderson, 2002; Hone, 2012). These ideas over time sometimes became established as received wisdom and were generally not subject to scrutiny or testing. Noted similarities between *Dimorphodon* and puffin rostra, as evidence of piscivory in the pterosaur, for example (see Section IV.2) (Seeley, 1901; Bakker, 1986), were overly simplified as the *Dimorphodon* rostrum is formed from bone whereas the *Fratercula* rostrum is formed from keratin and soft tissues (Ősi, 2011; Badikova & Dzerzhynsky, 2015). More sophisticated flight models indicate that *Dimorphodon* did not have the flight profile of an able fisher (Witton, 2008, 2015a), which has begun to cast doubt on this long‐untested hypothesis of diet for this pterosaur. Earlier flight models directly extrapolated pterosaur weights from modern birds with little account for the structural differences between avian and pterosaurian skeletons, with interpretations of pterosaur feeding ecology, and consequently diet, sometimes constructed from these models (Padian, 1980, 1983; Rayner, 1989; Hazlehurst & Rayner, 1992). Many later quantitative studies account for independent evolutionary histories and have produced strongly supported dietary hypotheses for some pterosaurs, including anurognathids (see Section IV.3) (Habib & Hall, 2012; Habib & Witton, 2013), and pteranodontids (see Section IV.9*a*) (Hone & Henderson, 2014; Habib, 2015). However, in other groups they have provided evidence for new diets, such as generalism in basal ctenochasmatoids (Henderson, 2018).

Current dietary diversities and evidential categories show that we currently lack reliable evidence to support dietary interpretations for many pterosaur groups, and this prevents us from teasing out the biological signals explaining pterosaur diet diversity and disparity. Greater use of robust, experiment‐led approaches, in tandem with other lines of evidence, is therefore needed to construct plausible dietary hypotheses in pterosaurs with low levels of dietary consensus and to test interpretations in pterosaurs with higher levels of consensus. This will help uncover biological signals for understanding pterosaur dietary evolution and for reconstructing Mesozoic ecosystems.

### (2) How appropriate are current methods for forming dietary hypotheses?

The range of methods employed in analysis of pterosaurs has proven highly successful in generating interpretations of diet. What is less clear, however, is how well they have performed in differentiating between alternative interpretations, and this partly explains why, for most pterosaur groups, multiple interpretations have been proposed. Greater use of analytical and experiment‐led methodologies (Veldmeijer *et al*., 2007; Padian, 2008a), along with careful experimental design and full appreciation of the methods and study taxa (Unwin & Henderson, 2002; Hutchinson, 2012) has the potential to improve both the rigour and consistency of dietary interpretations. No single method will provide complete understanding; application of multiple independent techniques is likely to yield the most robust interpretations.

#### (a) *Comparative anatomy*

Comparative anatomy is fundamental for analysis of homology and character evolution but has limitations as a tool for analysis of function and diet. Its application in this context is based on the assumption that convergence of morphological structures between extant and extinct taxa allows us to infer similar functional roles for structures in extinct taxa, including foraging and feeding ecology (Gould & Lewontin, 1979; Fisher, 1985; Purnell, 1999; Ferry‐Graham *et al*., 2002). In essence, interpretations are based on analogy (see Thomason, 1995).

There are a number of difficulties with this approach, not least of which is that many examples of its application can be criticised as ‘adaptationist’ (Gould & Lewontin, 1979; Fisher, 1985; Ferry‐Graham *et al*., 2002). Criticism is valid where structures are studied as single traits that are assumed to be optimised for their particular hypothesised function, without consideration of phylogenetic and fabricational constraints that limit organisms' capacity to adapt to selective forces (Seilacher, 1970; Gould & Lewontin, 1979). Similarly, for many structures it is probably unrealistic to assume that they are adapted to perform a single function, resulting in trade‐offs and a structure that is optimally adapted for neither role. The link between form and function is not always evident: teeth of one form can be applied to the consumption of a variety of foodstuffs, for example. This is what lies behind Liem's paradox, where organisms possess feeding‐related morphologies that are apparently specialised for particular food items, but whose typical diets are composed of other constituents (Robinson & Wilson, 1998; Binning, Chapman & Cosandey‐Godin, 2009). Conversely, traits that appear different can perform the same functions. Categorised as many‐to‐one mapping of form and function (Wainwright *et al*., 2005), this further undermines the value of analogy in interpreting function and diet. Unfortunately, many interpretations of pterosaur diet that draw on evidence of comparative anatomy fall into these traps. Most analyses focus exclusively on skulls, jaws or even individual teeth (Fastnacht, 2005; Ősi, 2011), and while not necessarily wrong, they often fail to account for the various constraints on optimal design and the possibility of many‐to‐one mapping.

The main issue is that hypotheses and interpretations generated through analogy and comparative anatomical approaches in themselves provide no mechanism for testing, and in some cases fail to pass the basic scientific criterion of testability. One interpretation of diet based on an extant functional analogue might conflict with another interpretation, equally well supported with a different analogue. Without employing methods in addition to comparative anatomy, such as functional morphological analyses, the only criteria upon which to evaluate such conflicting hypotheses are the ultimately subjective assessments of plausibility [see Purnell (1999) for discussions of similar issues in a different phylogenetic context].

#### (b) *Associations*

Despite the straightforwardness of using associations, deriving dietary interpretations from simple presence and/or absence of contemporaneous fossils amounts to little more than speculation. Likewise, solely examining depositional environments does not account for organism dispersal abilities and taphonomic biases such as carcass transportation (Veldmeijer *et al*., 2007; Witton & Naish, 2008; Tütken & Hone, 2010). Associations are nevertheless important for pterosaur research as they can serve as starting points for forming hypotheses when using experimental approaches.

#### (c) *Functional morphology*

**Figure 8:**
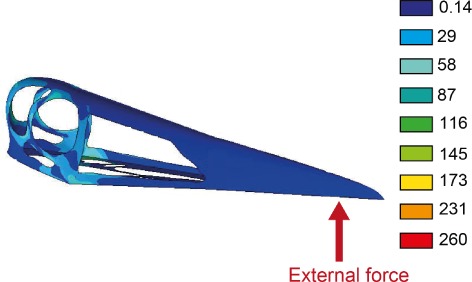
Finite Element Analysis output of a *Pterodactylus* skull in right lateral view with teeth absent. Around 100 N of external force, denoted by the red arrow, is applied to the skull anterior to simulate a bite. Colour deformation indicates Von Mises stress (MPa), with brighter colours denoting high stress as indicated in the key. N.B.: *Pterodactylus* bone strength was taken from human skull strengths. Adapted from Fastnacht (2005).

Quantitative methods have begun to shift the emphasis away from a largely comparative, analogy‐based framework towards a more hypothesis‐testing approach, but also have their own issues. For example, structural attributes, including bone strength, can never be directly measured from fossil material and must be inferred from extant analogues. This is especially problematic for taxa with no extant descendants, such as pterosaurs. Functional morphological models are therefore only as representative as the parameters set and assumptions made in the experimental design (Anderson *et al*., 2011). Some investigations into pterosaur biting behaviours, such as the FEA and cantilever analyses of Fastnacht (2005) (Fig. 8), used human bone strengths as substitutes. These issues can be mitigated through sensitivity analyses designed to test the impact of starting conditions, such as the mechanical properties of structures, on the outputs of biomechanical models (Bright & Rayfield, 2011). The mechanical properties of bone and skeletal structures for theses analyses can be measured in extant taxa using strain gauges, either in live feeding trials (*in vivo* data) (Porro *et al*., 2013), or in experimental application of external loads to the skulls of deceased organisms (*ex vivo* data) (Bright & Rayfield, 2011; Rayfield, 2011). Sensitivity analyses on *ex vivo* data from domestic pig (*Sus domesticus*) crania, for example, found that models which accounted for the heterogeneous nature of material properties in bone across the cranium exhibited fewest discrepancies with experimental outputs (Bright & Rayfield, 2011). Bone strength data can be subsequently extrapolated into models of extinct taxa, such as pterosaurs, and will improve our understanding of extinct functional behaviours (Degrange *et al*., 2010; Bright, 2014).

*In vivo* and *ex vivo* validation tests have also been performed on modern archosaurs including alligators (Porro *et al*., 2013) and ostriches (Rayfield, 2011), but further tests are needed on other archosaurs before these data can be confidently extrapolated to pterosaurs. Potential candidates include sea birds and piscivorous crocodilians as they have been suggested as pterosaur analogues (Seeley, 1901; Hazlehurst & Rayner, 1992; Witton, 2008; Bennett, 2014), and form an extant phylogenetic bracket around pterosaurs (Bennett, 1996; Nesbitt, 2011), with size and bone thickness differences between these taxa and pterosaurs taken into account. Adductor and neck musculatures could also be incorporated further to assess stress and strain distributions (Lautenschlager *et al*., 2013). This could help confirm whether pterosaurs procured and processed food items as hypothesised from earlier muscle reconstructions (Ősi, 2011; Pinheiro *et al*., 2014). Functional impacts of pterosaur rhamphothecas should also be investigated for relevant taxa because food items do not make direct contact with bone during feeding (Lautenschlager *et al*., 2013).

**Figure 9:**
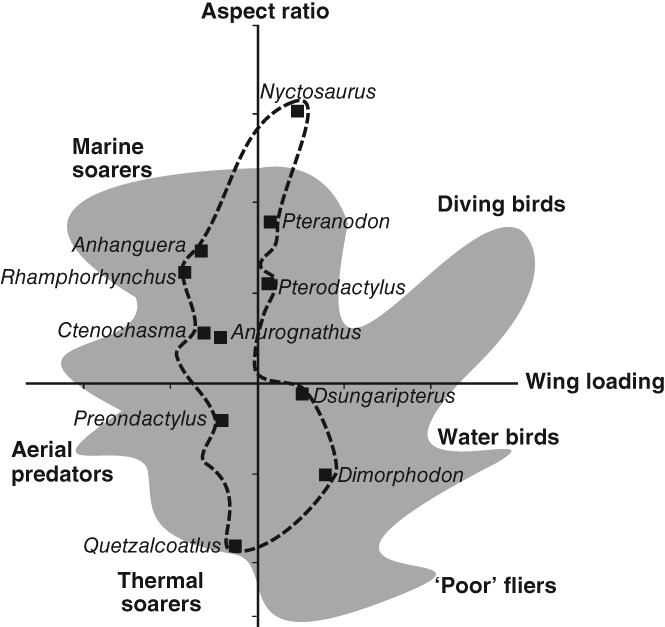
Ecomorphospace of modern birds (grey shading) and 19 sampled pterosaurs (black dashed area with selected genera) with respect to aspect ratio and wing loading, as deduced from mass estimations and bone strength analyses, to infer possible flight styles. Specified foraging ecologies (marine soarers, diving birds, water birds, ‘poor’ fliers, thermal soarers, aerial predators), refer to modern bird flight styles. Adapted from Witton (2013).

Understanding pterosaur locomotion, including terrestrial (walking), aerial (stall speeds) and aquatic‐based (diving and wading) behaviours can provide new constraints, or corroborate existing constraints, on possible foraging strategies and food‐acquisition behaviours. Locomotory models, however, are heavily influenced by mass estimations (Witton, 2008, 2015a; Henderson, 2010; Witton & Habib, 2010). Greater account needs to be made for the unique pterosaur body construction to allow pterosaur flight profiles to be more confidently placed within suitable biological contexts (Fig. 9). Humerus and femur structural strength models used for anurognathids (see Section IV.3) could be applied to other pterosaurs to investigate terrestrial behaviours (Habib & Hall, 2012; Habib & Witton, 2013). Terrestrial behaviours can also be partially verified through ichnofossil interpretations. Energy consumption modelling (see Section IV.8) should be used for pterosaurs with well‐constrained mass estimates to calculate energy expenditures of locomotory behaviours to assess energetic viabilities of different diets (Habib, 2015).

#### (d) *Content fossils*

Content fossils, although rare, are an important part of investigations into extinct diets (Wild, 1984; Hone & Faulkes, 2014; Hone *et al*., 2015a; Witton, 2018). Over‐reliance on further discoveries of these fossils, however, is ill advised. Issues include alternative explanations of content fossils as *post mortem* artefacts, with items introduced *via* water flows (Tweet *et al*., 2008), and incorrectly inferring extents of dietary specialism through preservation of items consumed almost immediately prior to death (Platt *et al*., 2013b; Davis & Pineda‐Munoz, 2016), and/or through small sample sizes (e.g. Szczepanski & Bengtson, 2014; see Section V.1). While careful consideration of specimen depositional environments should help account for *post mortem* artefacts, content fossil evidence can only ever indicate that a species sometimes consumed a particular food item and is therefore best interpreted in conjunction with other lines of evidence (Frey & Tischlinger, 2012).

#### (e) *Ichnofossils*

**Figure 10:**
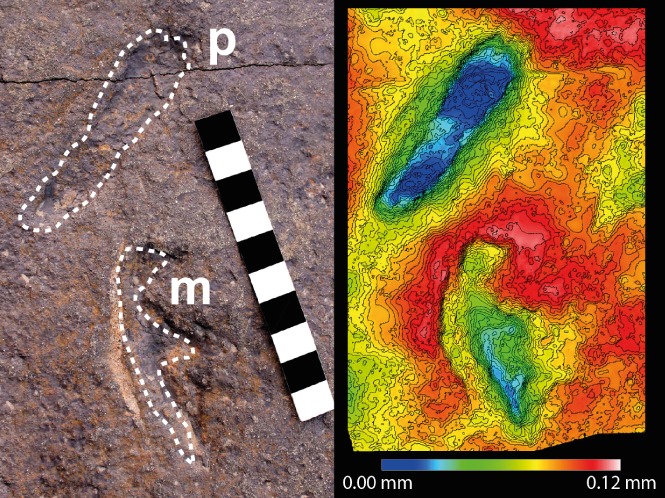
Left; outlined pterosaur *pes* (p; hindlimb) and *manus* (m; forelimb) impressions (Institute of Geology and Palaeontology, Linyi University, Linyi City, Shandong, China; LUGP3–001.2) from the Qugezhuang Formation, China. Scale bar, 10 cm. Right; 3D photogrammetric model of artificial casts of LUGP3–001.2. Blue denotes higher topographies, red‐white areas denote lower topographies. Topographical differences between the *pes* and *manus* impressions are potentially caused by variable substrate consistencies. Adapted from Xing *et al.* (2012).

Well‐preserved ichnofossils can indicate possible foraging and/or feeding behaviours such as swimming and sediment probing (Hwang *et al*., 2002). However, extrapolating inferred behaviours from trace fossils to contemporary pterosaurs does not guarantee those taxa were the perpetrators. Potential peck marks are yet to be verified as pterosaurian in origin. The absence of Late Triassic and Early Jurassic pterosaur ichnofossils may reflect an unknown preservation bias towards environments with softer sediments (Lockley & Wright, 2003; Witton, 2018), as opposed to air‐based feeding (Unwin, 2007). Ichnology is becoming more objective through 3D photogrammetry, which produces high‐resolution digital reconstructions *via* alignment of photographs taken from numerous angles (Fig. 10) (Falkingham, 2012; Breithaupt *et al*., 2015). 3D photogrammetry offers more‐effective ways of assessing ichnofossil perpetrators, locomotory behaviours and depositional environments (Fig. 10) (Xing *et al*., 2012; Breithaupt *et al*., 2015), which can aid in investigating diet.

#### (f) *Stable isotope analysis*

Analysis of stable isotopes provides an additional source of data that is independent of morphological evidence. It is commonly applied to extant vertebrates as a tool for dietary analysis, in many cases focusing on δ^15^N and/or δ ^13^C. In the context of extant animals, isotopic approaches to dietary reconstruction are not without methodological limitations (Nielsen, Popp & Winder, 2015; Nielsen *et al*., 2017). Analysis of δ^15^N, for example, provides a measure of the relative trophic position of a species within a specific trophic web, rather than food items consumed (Crawford, McDonald & Bearhop, 2008), and multiple dietary combinations can result in the same δ ^13^C and δ^15^N values (Caut, Angulo & Courchamp, 2009). Using isotopic evidence to infer diets in fossils is even more problematic. Some of the most informative stable isotopic analyses for dietary reconstruction, such as δ^15^N, are based on sampling tissues and biomolecules that do not preserve in typical fossils that are millions of years old (e.g. blood, muscle, hair, bone collagen). Analysis of carbon isotopes from tooth enamel is possible, but this is primarily used to infer the relative proportions of C_3_ and C_4_ plants consumed (see Davis & Pineda‐Munoz, 2016), so not useful for analysis of pterosaurs.

**Figure 11:**
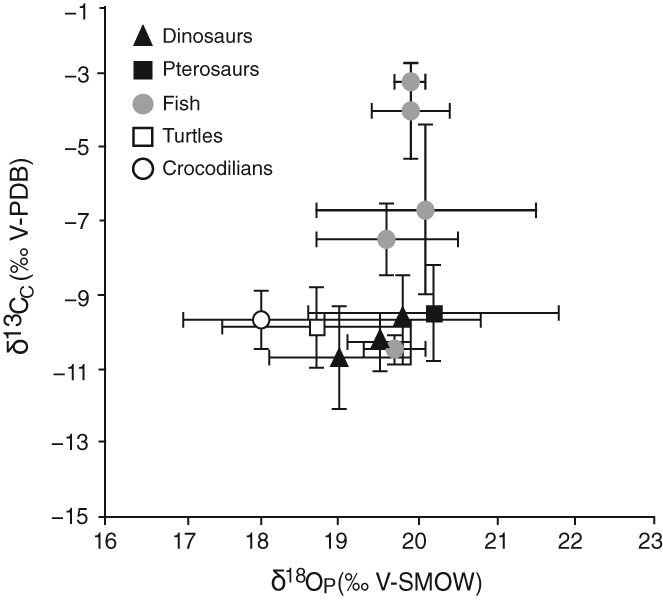
Stable isotope analysis of ornithocheirid teeth (black filled square), and terrestrial and aquatic vertebrates from the Upper Cretaceous Kem Kem beds, Morocco. Oxygen is expressed as δ^18^O *versus* standard mean oceanic water (V‐SMOW) and carbon is expressed as δ^13^C *versus* a marine carbonate (V‐PDB). The pterosaur δ^18^O range is generally similar to fish δ^18^O ranges (grey filled circles), although the pterosaur δ^13^C range is more similar to other vertebrates (dinosaurs, black filled triangles; turtles, open square; crocodilians, open circle). This suggests that Kem Kem pterosaurs consumed freshwater fish. Adapted from Amiot *et al.* (2010).

To date, there have been only a few investigations of stable isotopes in pterosaurs, primarily because sampling is destructive and well‐preserved pterosaur remains are comparatively rare. The analyses looked at δ^18^O and δ^13^C (Amiot *et al*., 2010; Tütken & Hone, 2010), which provide evidence of habitat rather than diet *per se*, i.e. signals of freshwater *versus* marine environments, for example (Fig. 11). Future work could focus on well‐preserved fragmentary, and thus expendable, material such as isolated teeth, to provide new dietary constraints for pterosaurs.

#### (g) *Quantitative 3D microtextural analysis of tooth microwear*

Only one study has examined dental wear patterns on pterosaur teeth and used this to infer aspects of diet and the material properties of food (Ősi, 2011). Dietary hypotheses from this study were constructed from observations of gross pterosaur tooth and wear pattern morphology using two‐dimensional (2D) images from scanning electron microscopy (SEM) (Ősi, 2011). Identifying and interpreting features in this way however, can be problematic because inter‐observer error rates are high and identification of wear features is influenced by the orientation of specimens relative to the electron beam and detector within the SEM (Ungar *et al*., 2003; Scott *et al*., 2005, 2006).

A more robust approach involves quantitative analysis of the sub‐micron scale 3D surface textures of teeth, known as dental microwear texture analysis (DMTA; Ungar *et al*., 2003; Scott *et al*., 2006). Microwear is produced on teeth when an organism feeds, as interactions with food items cause microscopic chipping and scratching on tooth surfaces (Rensberger, 1978; Walker, Hoeck & Perez, 1978; Teaford, 1988). As microwear formation is determined by the material properties of food, the technique provides direct evidence of the nature of what has been consumed, and analysis does not rely on assumptions of a close relationship between the morphology and inferred functions of teeth (Purnell, Seehausen & Galis, 2012; Daegling *et al*., 2013; Purnell & Darras, 2016). Because data acquisition is not operator dependent, DMTA avoids the inherent observer bias of 2D analyses. Most applications of DMTA focus on placental mammals with occlusal dentitions, but it is applicable to dietary analysis of older, more basal material, and to taxa with non‐occlusal dentitions (Purnell *et al*., 2012; Gill *et al*., 2014; Purnell & Darras, 2016). Its applicability to pterosaurs is the subject of on‐going investigation. Analysis of tooth surface textures in pterosaurs, when validated against those of extant taxa with known dietary differences, has the potential to provide robust tests of dietary hypotheses.

## VI. CONCLUSIONS

(1) A range of diets have been proposed for pterosaurs including insectivory, piscivory, carnivory, durophagy, filter‐feeding and generalism.

(2) Most pterosaur dietary interpretations are supported by qualitative evidence including comparative anatomy, associations, content fossils and ichnofossils with a minority supported by quantitative evidence from functional morphology and isotope analyses.

(3) Some pterosaur principal groups exhibit high levels of consensus regarding diet, supported by several evidential categories; others exhibit lower levels of consensus, with different interpretations inferred from conflicting evidence of the same categorical type, and typically poorly constrained analogy drawn from comparative anatomy.

(4) More speciose pterosaur groups exhibit higher diversity of hypothesised diets. Whilst this may reflect biological signals such as ecological radiations or niche partitioning, non‐biological causes, such as historical biases in the data, quality of the fossil record and research intensity are more likely. These biases mean it is currently difficult to reliably test the hypothesis that the apparent patterns of pterosaur dietary diversity and disparity are biologically controlled.

(5) Examining patterns of dietary diversity using different phylogenies reveals higher consensus among monofenestratan groups and lower consensus in non‐monofenestratan groups. Better resolution on the membership of pterosaur groups would assist in uncovering true diets for respective groups.

(6) Numbers of pterosaur publications per year and dietary interpretations do not correlate through time. The majority of interpretations were proposed in the 21st century. The almost exponential rise in the number of publications containing dietary interpretations since the 1980s coincides with discoveries of new Lagerstätten and other exceptionally preserved sites, as well as applications of new techniques to pterosaurs.

(7) Many dietary interpretations are based on simple extrapolations and comparisons with modern biology with little scope for testing. Qualitative methods can serve as starting points for generating hypotheses, but quantitative tests provide more robust analyses and insights into dietary diversity, evolution and the ecological roles of pterosaurs. Improvements to current methods and application of novel methods to pterosaurs will provide better constraints on diets in pterosaurs with low levels of consensus, and better tests of dietary hypotheses in pterosaurs with high levels of consensus. This will allow reliable investigations into possible biological signals behind pterosaur dietary diversity and disparity, which will allow greater understanding of pterosaur dietary evolution and facilitate reconstructions of Mesozoic ecosystems.

## Supporting information


**Appendix S1.** Breakdowns of all pterosaur dietary interpretations, detailing dietary and evidential category assortments, for each of the Unwin, Kellner and Andres phylogenies. The Kellner and Andres pterosaur phylogenies (see online Figs S1 and S2, respectively) and dietary interpretations for each pterosaur principal group (see online Figs S3 and S4, respectively) are also included. Further information on phylogenies and the full list of 180 pterosaur species assorted into principal groups from the Unwin phylogeny is also provided.
